# Raman focal point on Roman Egyptian blue elucidates disordered cuprorivaite, green glass phase and trace compounds

**DOI:** 10.1038/s41598-022-19923-w

**Published:** 2022-09-16

**Authors:** Petra Dariz, Thomas Schmid

**Affiliations:** 1 Bern University of the Arts, 3027 Bern, Switzerland; 2 Bundesanstalt für Materialforschung und -prüfung (BAM), 12489 Berlin, Germany

**Keywords:** Mineralogy, Analytical chemistry

## Abstract

The discussed comparative analyses of Roman Imperial pigment balls and fragmentary murals unearthed in the ancient cities of Aventicum and Augusta Raurica (Switzerland) by means of Raman microspectroscopy pertain to a predecessor study on trace compounds in Early Medieval Egyptian blue (St. Peter, Gratsch, South Tyrol, Northern Italy). The plethora of newly detected associated minerals of the raw materials surviving the synthesis procedure validate the use of quartz sand matching the composition of sediments transported by the Volturno river into the Gulf of Gaeta (Campania, Southern Italy) with a roasted sulphidic copper ore and a mixed-alkaline plant ash as fluxing agent. Thus, the results corroborate a monopolised pigment production site located in the northern Phlegrean Fields persisting over the first centuries A.D., this in line with statements of the antique Roman writers Vitruvius and Pliny the Elder and recent archaeological evidences. Beyond that, Raman spectra reveal through gradual peak shifts and changes of band width locally divergent process conditions and compositional inhomogeneities provoking crystal lattice disorder in the chromophoric cuprorivaite as well as the formation of a copper-bearing green glass phase, the latter probably in dependency of the concentration of alkali flux, notwithstanding that otherwise solid-state reactions predominate the synthesis.

## Introduction

During the Roman period Egyptian blue was circulated throughout the Empire in the quasi standardised form of small balls of around 15 to 20 mm in diameter, thus the painter defined the respective grain size and by that the shade of blue and the covering capacity of the ground up artificial pigment himself^1,2^. In the first century B.C. Vitruvius provided the following guidance for its preparation in his architectural textbook *De architectura libri decem* (Liber VII, Caput XI), leaving out any details on quantities and processing temperature: “The recipes for [sky] blue were first discovered in Alexandria, and subsequently Vestorius began to manufacture it in Puteoli as well. […] Sand is ground with flower of natron […] so finely that it almost becomes like flour. Copper [ore], broken by coarse files until it is like sawdust, is sprinkled with this sand until it clings together. Then it is formed into balls by rolling it between the hands and bound together to dry. Once dry, the balls are put into a ceramic pitcher, and the pitchers are put into a kiln”^3^. In view of archaeological evidence and the concordant information given by Vitruvius as well as Pliny the Elder (first century A.D.)^4^, current research assumes a monopolised production site in the area of the ancient cities of Cumae and Liternum (Gulf of Pozzuoli, Campania, Southern Italy), whereas manufacture in Central Europe is excluded due to a very probable lack of technological abilities^5–10^. According to modern laboratory experiments, Egyptian blue is synthesised from a raw material blend of quartz sand, limestone, sulphidic copper or copper carbonate ore and alkali flux in the form of either natron or ash from halophytes (salt plants) at temperatures between 850 and 1000 °C under oxidising conditions^9,11–16^.

**Figure 1:**
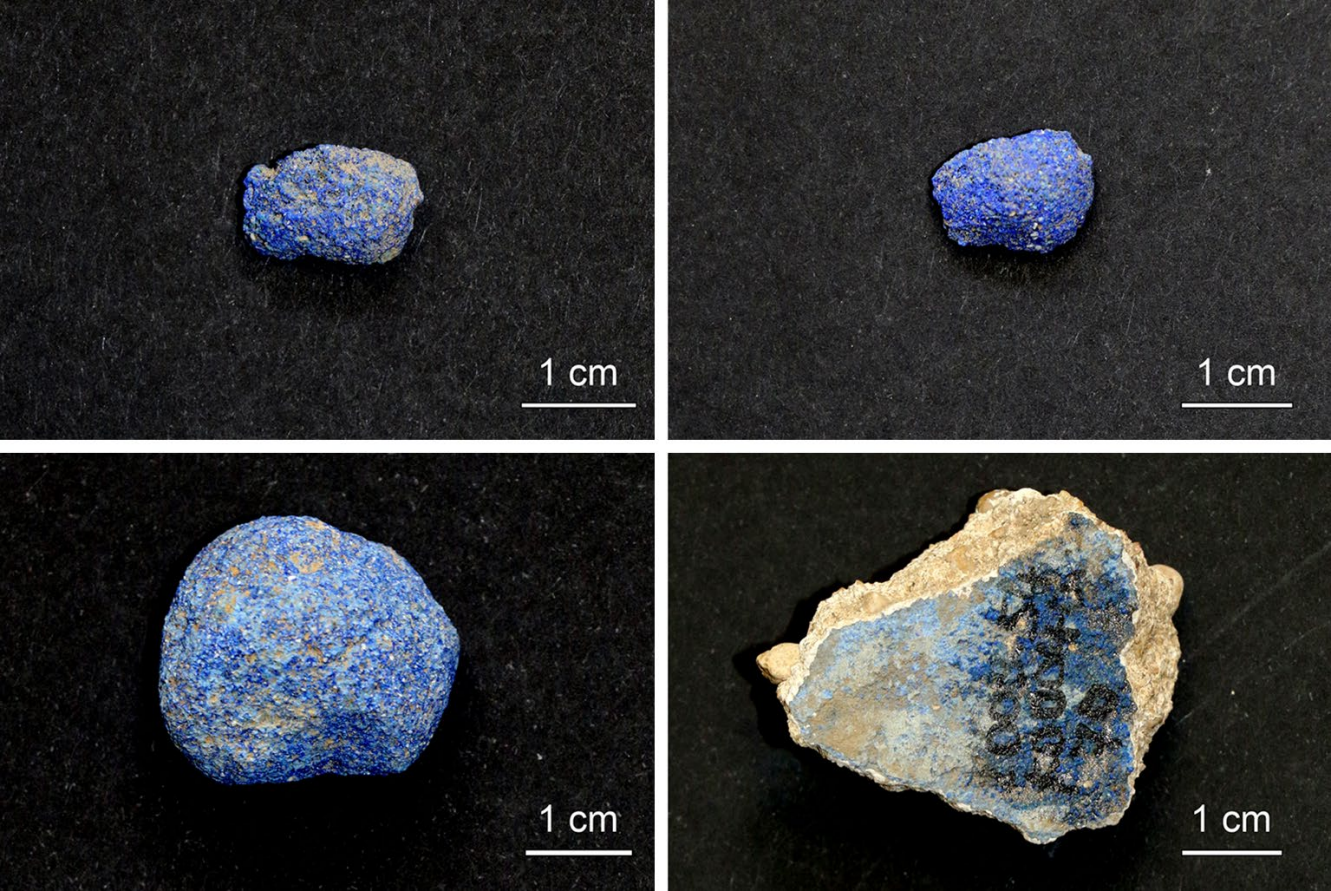
Egyptian blue pigment balls and mural fragment unearthed in the archaeological remains of the ancient Roman cities of Aventicum (top) and Augusta Raurica (bottom).

Only recently, a study on a monochrome blue mural fragment belonging to the Early Medieval church of St. Peter above Gratsch (South Tyrol, Northern Italy, fifth/sixth century A.D.) by means of area-covering Raman microspectroscopic imaging resulted in the identification of 26 minerals down to the sub-permille level in addition to the chromophoric cuprorivaite CuCaSi_4_O_10_—an assemblage suggestive of type and provenance of the raw materials and of chemical reactions occurring during pigment manufacture and application as well as ageing of the pictorial layer^17^. Especially some accessory minerals attributable to the quartz sand, which survived processing without thermal alterations, were indicative of an import of the Egyptian blue in question from the northern Phlegrean Fields in Campania. As detailed below, analogous analyses of pigment balls and of a fragment of a wall painting unearthed in the archaeological remains of the ancient Roman cities of Aventicum and Augusta Raurica (Switzerland) (Fig. 1) further extended the plethora of hitherto uncovered trace compounds and revealed, beyond that, particularities concerning the formation of crystalline and amorphous phases or the thermal history of the artificial blue, respectively.

## Samples

Table S1 in the Supplementary Information provides an overview of the Egyptian blue samples analysed in the present and predecessor paper^17^. The pigment balls under study (Fig. 1) are dated via adjoining archaeological finds, especially ceramics, to the second or third quarter of the first century A.D. (Roman Colonia Augusta Raurica) and the second half of the first century A.D. or the beginning of the second century A.D. (Aventicum, principal place of the Roman Civitas Helvetiorum). The monochrome blue mural fragment (excavated in the remains of the city of Augusta Raurica) in turn is assigned to the first half of the third century A.D.

The modern Egyptian blue used as reference material for Raman experiments is part of the range of re-enacted historical pigments of the colour mill Kremer Pigmente (Aichstetten, Germany).

## Methods

### Raman microspectroscopic imaging

Raman spectra were acquired using a Horiba JobinYvon Labram HR800 Raman microscope with 532 nm continuous-wave laser excitation (diode-pumped solid-state laser, 40 mW maximum power at the sample surface, reduced to 20 mW by a neutral density filter). The laser light was focused onto the sample surface and the reflected and/or scattered light was collected in upright configuration by using a 50×/N.A. = 0.55 long-working-distance microscope objective (with N.A. denoting the numerical aperture) leading to a focus diameter of approximately 1.2 µm. Dispersion of the Stokes-Raman-scattered light in a 800 mm spectrometer was carried out with a 300 mm^−1^ grating, and spectra were detected by a Peltier cooled (− 60 °C) charge coupled device (CCD) Syncerity camera (Horiba JobinYvon) having 1024 pixels along the wavenumber axis, resulting in spectra ranging from approx. 70 cm^−1^ to 3250 cm^−1^ with a spectral resolution of 3.7 cm^−1^ to 2.6 cm^−1^ per CCD pixel. Raman maps were gathered by software-controlled (Horiba JobinYvon LabSpec 6) stepwise movement of the sample stage through the laser focus with a step size of 1 µm. Typical acquisition time per pixel or spectrum, respectively, was 0.5 s with 10 to 40 accumulations, chosen depending on the signal to noise ratio and available measurement time. Single spectra, acquired independently of mappings, were typically measured within 1 min split into several accumulations (e.g., 6 × 10 s). See Ref.^18^ for further specifics of the employed instrument and an introduction to Raman microspectroscopic imaging, and Ref.^17^ for details on the optimisation of the measurement parameters adopted from the predecessor study on trace compounds in Early Medieval Egyptian blue. As the conditions cannot be adjusted to every mineral individually, the chosen irradiance reflects a compromise between sensitivity and non-destructiveness. Therefore, thermal conversion of coloured sulphides and oxysalts cannot be ruled out completely (see the section ‘Contaminations from adherent soil minerals’ below as well as the Supplementary Information of the predecessor study^17^ and references therein), which was considered in the interpretation of the results.

Measurement areas were randomly selected on the surfaces of the four samples. Mapping sizes were chosen depending on local sample roughness and ranged from 28 × 40 to 161 × 141 pixels. For each sample 12 to 16 Raman maps were acquired within a typical measurement time of 50 h each (when assuming 100 × 100 pixels and 36 × 0.5 s per pixel as typical mapping conditions). Altogether, 100,016 (pigment ball Aventicum forum; see top-left image in Fig. 1), 100,318 (pigment ball Aventicum insulae 15; Fig. 1, top right), 101,091 (pigment ball Augusta Raurica) and 99,885 spectra (mural fragment Augusta Raurica), respectively, were collected. These 401,310 spectra were evaluated by using own (T.S.) LabView-based (National Instruments, Austin, TX, USA) software developed for analysing Raman maps and enabling the calculation of two-dimensional distributions of baseline-corrected peak intensities of each Raman band found in a dataset and extracting their individual spectra. The latter were assigned to mineral phases by comparison with reference data from the RRUFF spectral library (https://rruff.info)^19^ or from the literature (see Figs. S3–S29, S35, S36, S42, S46, S49, S50, S53 and S54 in the Supplementary Information).

### Raman spectroscopy for analysing pyrometamorphic conversions

For simulating the effect of heat in an ancient furnace onto selected mineral phases, some preliminary in situ Raman measurements were carried out by employing a TS-1500 heating stage from Linkam Scientific Instruments Ltd. (Redhill, Surrey, UK) with a T96-LinkPad controller, placed under the same microscope objective with approx. 1 cm working distance mentioned above. Overall, the same typical measurement parameters were used. Such temperature-dependent experiments began with the measurement of the room-temperature spectrum of the sample in the 7 mm diameter crucible of the heating stage, followed by heating to a selected temperature with the highest possible rate of 200 K/min that was held constant for 5 min. Subsequently, a Raman spectrum was acquired for checking purposes (data not shown, as not relevant for the study at hand) and the sample was allowed to cool back to room temperature for gathering a Raman spectrum or map of the heat-treated material. The next temperature step was chosen, and the described procedure was repeated. The monitored pathways of such pyrometamorphic transformations are documented in Figs. S41–S48 and S52 in the Supplementary Information. The numbers given in Figs. S45 and S52 are based on a number of n measurements from Raman maps and represent mean values ± standard deviations.

### Peak fitting procedures applied in the evaluation of Raman spectra

Synthesis conditions of Egyptian blue and heat treatment of selected minerals have shown to significantly influence the widths and in some cases the centre wavenumbers of their Raman bands. For their exact determination with a resolution of approximately one order of magnitude better than in the raw spectroscopic data, according to the procedures described in Ref.^20^, individual Raman bands were fitted with Lorentzian functions, usually by employing the Levenberg–Marquardt algorithm provided by the software Origin 2020 (OriginLab Corp., Northampton, MA, USA). This applies to the spectra of crystalline phases shown in Figs. S32, S41, and S51, in the Supplementary Information as well as to the deconvolutions of glass spectra into individual peaks displayed in Figs. S39, S40, S44 and S48. The large dataset from mapping experiments presented in Fig. S33 was evaluated by the own LabView-based software mentioned above, enabling automated fitting within selected wavenumber range fractions of all Raman spectra of a whole map. (Only peaks with a baseline-corrected height exceeding a preselected threshold intensity were included in the evaluation). Here, the band widths were established by Lorentzian fitting using a Trust Region (Dogleg) algorithm, while for the determination of exact band positions a Gaussian Levenberg–Marquardt peak fitting was applied, because providing more stable results and because the identification of peak centres is less sensitive to the matching of the shapes of measurement data and fit functions. The Raman maps acquired for determination of the means and standard deviations displayed in Figs. S45 and S52 were also evaluated by Lorentzian fitting using the LabView software. The Raman band widths given in the Supplementary Information are intended to only show general trends and may vary when reproduced with different Raman instruments. Ref.^20^ provides strategies for correcting instrument-dependent band broadening; the band width data presented here correspond to the uncorrected ‘Horiba 532 nm’ dataset there.

### Light microscopy

**Figure 2:**
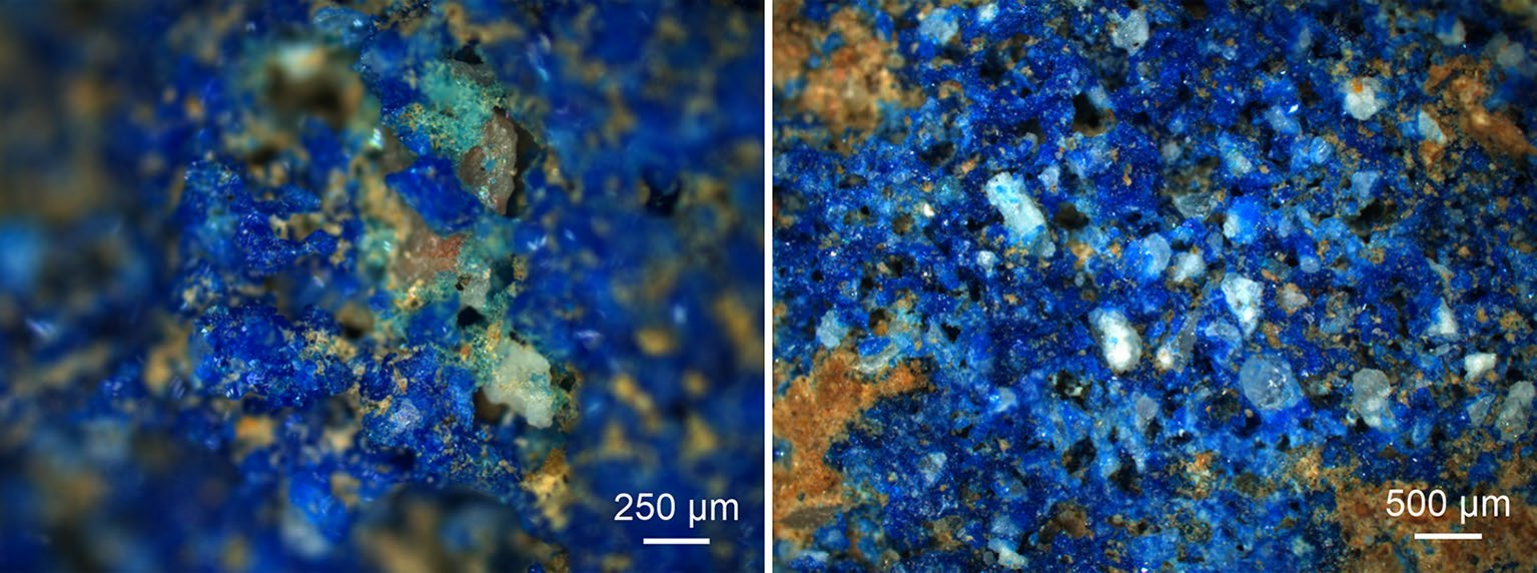
Darkfield light microscopy images of pigment balls unearthed in Aventicum (left; insulae 15) and Augusta Raurica (right). The left micrograph shows the green glass phase found as a by-product of the synthesis. Brown particles present in both images are due to adherent soil from the excavation sites (see Fig. S1 in the Supplementary Information for further micrographs).

The micrographs shown in Fig. 2 and Fig. S1 in the Supplementary Information were captured using a Zeiss AxioScope A.1 MAT with an AxioCam MRc Rev.3 camera in upright configuration with darkfield illumination.

## Results and discussion

### Mineralogy of the quartz sand

**Figure 3:**
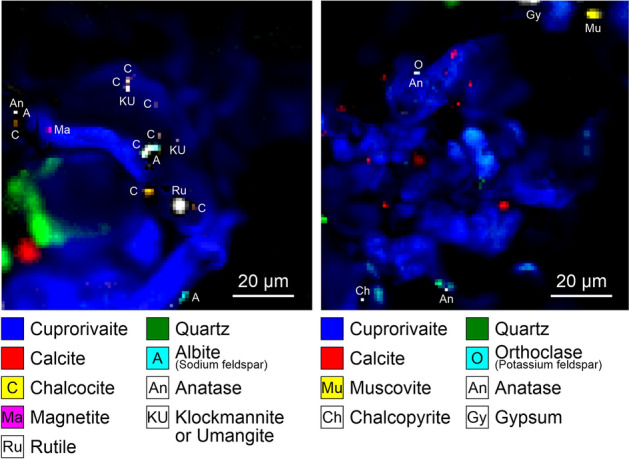
Selected Raman map of the pigment ball from Augusta Raurica (left) with chalcocite and accessories of the sulphidic copper ore. The paint layer of the mural fragment from the same excavation site (right) is contaminated with soil minerals, namely muscovite. This Raman map also contains the signature of disordered cuprorivaite, which is shown and discussed in detail in the Supplementary Information (Fig. S34; see Fig. S2 for further Raman maps).

Evaluation of available descriptive literature and data provides the following mineralogical composition of the “sand on a coast of six miles in length between Cumae and Liternum”, i.e. of sediments transported by the Volturno river into the Gulf of Gaeta (Campania, Southern Italy): The carbonate-bearing coastal sands are characterised by impurities in the form of feldspars (potassium feldspar KAlSi_3_O_8_, hyalophanes﻿ (K,Ba)Al(Si,Al)_3_O_8_, albite NaAlSi_3_O_8_, plagioclase), iron-rich augite (Ca,Na)(Mg,Fe,Al,Ti)(Si,Al)_2_O_6_, diopside CaMgSi_2_O_6_ (or salite Ca(Mg,Fe)Si_2_O_6_), hornblende and volcanic rock fragments, as well as natural glass and sporadic accessory minerals such as apatite Ca_5_(PO_4_)_3_(F,OH), biotite K(Mg,Fe)_3_(Si_3_Al)O_10_(OH,F)_2_, rutile TiO_2_, ilmenite FeTiO_3_, sphene CaTiSiO_5_, garnet (i.e. andradite Ca_3_Fe_2_Si_3_O_12_), magnetite Fe_3_O_4_, hematite α-Fe_2_O_3_, spinel and zircon ZrSiO_4_. Source rocks of the carbonates (predominantly calcite CaCO_3_, rarely dolomite CaMg(CO_3_)_2_) are the carbonate/siliciclastic successions of the Apennine chain^21–26^. A matching assemblage of trace compounds—at this time with the exception of hornblende, dark mica, ilmenite and sphene—was detected by means of Raman microspectroscopy (Fig. 3, Fig. S2) on the pigment balls unearthed in the ancient Roman cities of Aventicum and Augusta Raurica (Table 1), this in consistency with the relevant properties of the Early Medieval Egyptian blue applied in St. Peter above Gratsch studied recently^17^. In Switzerland occurrences of relatively pure quartz sand are concentrated in Rhaetian successions and pockets or basins of the Bean Ore Formation in the Jurassic, in particular in the Basle, Solothurn and Bernese Jura, and in littoral accumulations of the Marine Molasse in the Central Plateau (for example near Benken and Wildensbuch in the Zürcher Weinland). Seen their mineral constituents, these deposits can be excluded as raw material source for the manufacture of the blue pigment balls under study just as river sands or sands from local moraines encompassing heterogeneous rock types outcropping in the Swiss Alps and the Swiss Plateau^27–32^.

**Table 1:** Plethora of minerals identified in the Roman Imperial pigment balls and fragmentary wall paintings under study as well as in the monochrome blue paint layer of an Early Medieval mural fragment originating from St. Peter ob Gratsch (South Tyrol, Italy)^17^ by Raman microspectroscopy.

Mineral phase	Formula	Source/interpretation	Roman Imperial	Early Medieval
Cuprorivaite	CaCuSi_4_O_10_	Synthesis	X	X
Green Cu-glass		Synthesis	X	
Wollastonite	CaSiO_3_	Synthesis	X	
Cristobalite	SiO_2_	Synthesis/quartz sand	X	X
Silicate glass		Synthesis/quartz sand		X
Aegirine	NaFeSi_2_O_6_	Quartz sand	X	X
Andradite (garnet)	Ca_3_Fe_2_Si_3_O_12_	Quartz sand	X	
Augite-diopside	(Ca,Mg,Fe)_2_Si_2_O_6_	Quartz sand	X	X
Dolomite	CaMg(CO_3_)_2_	Quartz sand	X	X
Feldspars	M(I)_x_M(II)_1−x_Al_2−x_Si_2+x_O_8_	Quartz sand	X	X
Quartz	SiO_2_	Quartz sand	X	X
Rutile	TiO_2_	Quartz sand	X	
Zircon	ZrSiO_4_	Quartz sand	X	
Calcite	CaCO_3_	Quartz sand/carbonation	X	X
Aragonite	CaCO_3_	Carbonation		X
Anatase	TiO_2_	Quartz sand/copper ore	X	X
Apatite	Ca_5_(PO_4_)_3_(F,OH)	Quartz sand/copper ore	X	X
Brookite	TiO_2_	Quartz sand/copper ore	X	
Hematite	Fe_2_O_3_	Quartz sand/copper ore	X	X
Magnetite	Fe_3_O_4_	Quartz sand/copper ore	X	X
Arsenolite	As_2_O_3_	Copper ore	X	
Basic copper arsenate	Cu_x_(AsO_4_)_y_(OH)_2x-3y_	Copper ore	X	X
Cassiterite	SnO_2_	Copper ore		X
Chalcocite	Cu_2_S	Copper ore	X	X
Chalcopyrite	CuFeS_2_	Copper ore	X	
Cinnabarite	HgS	Copper ore	X	
Copper oxide	Cu_x_O	Copper ore	X	X
Eskolaite	Cr_2_O_3_	Copper ore		X
Greenockite	CdS	Copper ore	X	
Jacobsite	MnFe_2_O_4_	Copper ore	X	X
Kësterite-stannite group	e.g., Cu_2_(Zn,Fe)SnS_4_	Copper ore	X	
Klockmannite or umangite	CuSe or Cu_3_Se_2_	Copper ore	X	
Lead oxide	PbO_x_	Copper ore		X
Lead stannate or antimonate	Pb_2_SnO_4_ or Pb_2_Sb_2_O_7_	Copper ore	X	
Malayaite	CaSnOSiO_4_	Copper ore		X
Natrojarosite	NaFe_3_(SO_4_)_2_(OH)_6_	Copper ore		X
Osarizawaite	PbCuAl_2_(SO_4_)_2_(OH)_6_	Copper ore		X
Phoenicochroite or cochroite	Pb_2_(CrO_4_)O or PbCrO_4_	Copper ore	X	
Arcanite	K_2_SO_4_	Flux	X	
Bobierrite	Mg_3_(PO_4_)_2_·8H_2_O	Flux	X	
Epsomite	MgSO_4_·7H_2_O	Flux	X	
Morinite	NaCa_2_Al_2_(PO_4_)_2_(OH)F_4_·2H_2_O	Flux	X	
Polyhalite	K_2_Ca_2_Mg(SO_4_)_4_·2H_2_O	Flux	X	X
Syngenite	K_2_Ca(SO_4_)_2_·H_2_O	Flux		X
Thénardite or aphthitalite	Na_2_SO_4_ or (K,Na)_3_Na(SO_4_)_2_	Flux	X	
Weddellite (oxalate)	Ca(C_2_O_4_)·2H_2_O	Ageing		X
Amorphous carbon	C	Underpainting		X
Gypsum	CaSO_4_·2H_2_O	Adherent soil	X	
Muscovite	KAl_2_(Si_3_Al)O_10_(OH,F)_2_	Adherent soil	X	
Stilpnomelane	K(Fe,Mg,Al)_8_(Si,Al)_12_(O,OH)_27_·2H_2_O	Adherent soil	X	

### Constituents and accessory minerals of the copper ore

The observation of remnant chalcocite Cu_2_S and chalcopyrite CuFeS_2_ points to the use of a sulphidic copper ore as copper source for the synthesis of Egyptian blue. These two most common copper minerals are accompanied as usual^33^ by various sulphides (kësterite Cu_2_(Zn,Fe)SnS_4_ and other members of the stannite group, cinnabarite HgS, greenockite CdS), selenides (klockmannite CuSe or umangite Cu_3_Se_2_), arsenates (arsenolithe As_2_O_3_, basic copper arsenate Cu_x_(AsO_4_)_y_(OH)_2x−3y_), chromates (phoenicochroite Pb_2_(CrO_4_)O or crocoite PbCrO_4_) and oxides. Depending on the temperature resistance, in some cases only the Raman microspectroscopic detection of oxidation or (pyrometamorphic) reaction products was feasible due to the essential roasting of the sulphidic copper ore preceding the pigment production or due to the synthesis accomplished in an oxidising furnace atmosphere, though also the presence of secondary minerals originating from the oxidation zone of the copper deposit cannot be ruled out (this applies for instance to cuprite Cu_2_O and lead stannate/lead tin yellow I Pb_2_SnO_4_ or lead antimonate/Naples yellow/oxyplumboroméite (the former bindheimite) Pb_2_Sb_2_O_7_^34–36^). The identified oxides of the spinel group—mixed crystals aside from the end members magnetite Fe_3_O_4_ and jacobsite MnFe_2_O_4_—might be assigned as subordinate minerals to the quartz sand as well^21–26^. In summary, the evidenced accessories do not embody any distinguishing feature for provenancing the processed copper ore.

### Type of alkali flux

Usually, the ratios of Na_2_O/K_2_O, Na_2_O/MgO and Na_2_O/CaO established in ancient Egyptian blue by means of elemental analysis are employed to identify—analogous to contemporary glass or faience glaze—the type of alkali flux in the raw material mixture, as they are affected by impurities in either natron (i.e. a polyphase geogenic evaporite consisting of carbonates, bicarbonates, sulphates and chlorides of sodium) or ash of halophytes. All values are significantly lower in plant ash, although sodium, potassium, magnesium and calcium originating from natural associated minerals of the quartz sand (e.g. alkali feldspar or its alteration products like kaolinite Al_2_Si_2_O_5_(OH)_4_, limestone, mollusc shells, etc.) can also influence the concentration ratios of these chemical elements (recalculated into oxidic form according to convention), thus potentially leading to incorrect conclusions. Beyond that, the composition of plant ash and glass or glaze, respectively, differs, since any sulphates or chlorides present in the flux form a separate salt melt, the so called *galle*, whereas more reactive (hydrogen) carbonates, sulphites, sulphides and hydroxides are more easily incorporated in the melt^37–41^. An ion exchange between coexisting salt and silicate melt and similar processes during the synthesis of Egyptian blue can be assumed on condition of melt formation^39^, but in contrast to the manufacture of glass, the separation of unreacted salts is not part at least of the procedure described by Vitruvius^3^. Notwithstanding the in the present case unfeasible elemental or phase quantification, we interpret the main detection of sulphates (arcanite K_2_SO_4_, thénardite Na_2_SO_4_ and/or aphthitalite (K,Na)_3_Na(SO_4_)_2_, epsomite MgSO_4_·7H_2_O, polyhalite K_2_Ca_2_Mg(SO_4_)_4_·2H_2_O) and particularly phosphates (bobierrite Mg_3_(PO_4_)_2_·8H_2_O, morinite NaCa_2_Al_2_(PO_4_)_2_(OH)F_4_·2H_2_O) by chemical imaging via Raman microscopy as indicative of the use of a fluxing agent in the form of soda-rich or mixed-alkaline ash derived from salt tolerant plants of the genera Salsola or Suaeda (both belonging to the so-called saltworts) from the amaranth family (Amaranthaceae) such as the glasswort *Salsola kali* (or synonym *Kali turgidum*) flourishing on the Mediterranean coasts^38,40,42^. According to the relevant literature, variable amounts of phosphate (up to 2 wt%) in the glass phase of Egyptian blue samples originating from tombs and temples dated to the fifth dynasty﻿ of the Old Kingdom till the Roman time provide evidence for the application of plant ash fluxes rather than alkali salts^12,25,43^—this because geogenic evaporitic natron is virtually free from phosphate (and potassium) salts, whereas phosphate forms a chemical main component of biogenic native plant ash and still a minor constituent of its extract^42,44–51^.

### Thermal history of the pigment balls

**Figure 4:**
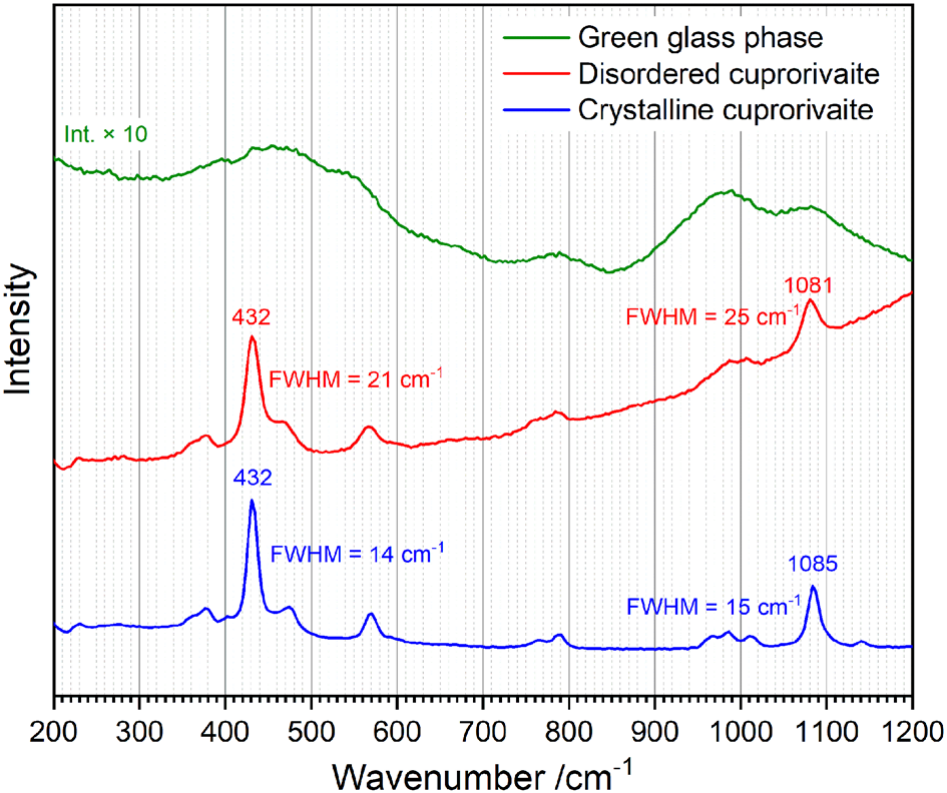
Comparison of the Raman spectra of the green glass phase (intensity × 10; acquired on the pigment ball unearthed in Augusta Raurica) and disordered as well as crystalline cuprorivaite (both from the same Raman map of the pictorial layer of the mural fragment excavated in the remains of Augusta Raurica).

Detailed evaluation of the comprehensive spectroscopic data of cuprorivaite (see Fig. S29 in the Supplementary Information) acquired within this study revealed individual crystals with lattice disorder through comparable deviations in Raman spectra, i.e. changes of band widths accompanied by gradual peak shifts reflecting differences in crystallinity when considering only the ancient Egyptian blue, but also when confronting Roman Imperial with modern (Kremer Pigmente) sample material (see Fig. 4). Such band width effects can be explained with crystal lattice defects as well as the extent of the relative surface area, as experimentally demonstrated for the example of thermal anhydrite CaSO_4_ grains in high-fired medieval gypsum mortar by combined Raman, X-ray diffraction (XRD) and Brunauer–Emmet–Teller (BET) measurements^52^; in other words, a highly crystalline material, characterised by sharp Raman bands, consists of relatively large crystallites exhibiting only few lattice defects. A look into the crystal structure of cuprorivaite (see Fig. S30 in the Supplementary Information) enables access to the interpretation of its spectra and the monitored variations: Rings of four silicon and four oxygen atoms (O_ring_) are connected by bridging oxygens (O_bridge_) and alternatingly arranged within the sheets of this phyllosilicate. Terminal Si–O^−^ groups (O_term_) coordinate Cu^2+^ ions occupying vacancies between the four-membered rings within the layers as well as the Ca^2+^ ions interconnecting the layers. The most prominent Raman mode of cuprorivaite at approx. 432 cm^−1^ (due to orientation/polarisation effects not always the strongest peak^11^) is assigned by Pietro Baraldi et al.^14^ to a combination of ring deformation modes detected at 427 cm^−1^ in the structural analogue gillespite BaFeSi_4_O_10_ by David McKeown and Michael Bell^53^ (see Table S3 for details on the assignments of Raman bands). (A breathing motion of the O_ring_ atoms, which owing to the relatively weak bonds to the Cu^2+^ and Ca^2+^ ions also involves slight motions of the Si–O_term_ groups, might contribute as well). The second strong band at approx. 1087 cm^−1^ is because of a localised stretching motion along the Si–O_bridge_–Si axes (see Fig. S31, Table S2 for all experimentally determined Raman modes of cuprorivaite).

#### Disordered cuprorivaite

While in disordered cuprorivaite all Raman bands broaden, the most pronounced band shift occurs in the case of the Si–O_bridge_–Si stretch vibrational mode between approx. 1081 cm^−1^ (lowest crystallinity) and 1088 cm^−1^ (highest crystallinity) (see Figs. S32a, S33a). Such large band shift is far beyond the effect typically observed for strain in crystalline materials^54^ of < 1 cm^−1^ and is either due to exchange of ions having different masses or due to a significant variation in force constants. The first possibility can be ruled out, as the same peak shifts also occur when measuring modern cuprorivaite synthesised from pure starting materials (see Figs. S32b, S33a). Thus, the downshift of the Si–O_bridge_–Si stretch frequency in disordered cuprorivaite can be explained by a significant weakening of the interconnections within the silicate sheets. This hypothesis is confirmed by relatively strong shifts in the same direction of the bands at 473 cm^−1^ (shoulder of the 432 cm^−1^ peak) and 570 cm^−1^ (values corresponding to highly crystalline cuprorivaite), as the vibrations at both wavenumbers include bending motions of the Si–O_bridge_–Si bridges: the first represents a Si–O_term_/Si–O_bridge_ rocking, the latter a O_term_–Si–O_bridge_ bending mode. Furthermore, another slight wavenumber downshift indicating defects of the cuprorivaite structure was found for the peak at 114 cm^−1^, representing the motion of Ca^2+^ ions relative to the silicate structure^14,53^, which can be interpreted as weakening of the interactions between the sheets (see Fig. S32). In contrast to these modes, the band position at 432 cm^−1^ is almost unaffected by cuprorivaite’s degree of imperfection or Raman band widths, respectively (see Fig. S33b). (A slight increase of binding strength within the rings in disordered cuprorivaite is the necessary consequence of the weakening of the bridging bonds interconnecting these structural elements).

In summary, these spectroscopic properties elucidate that in disordered cuprorivaite mainly the layer structure is not fully developed and characterised by weakened intra- and inter-sheet bonding due to insufficient reaction time, while the four-membered silicate rings are established like in the crystalline form. Because of incomplete conversion, a considerable amount of cuprorivaite exhibiting lattice disorder was found colocalised with remnant quartz in the Roman Imperial pigment balls (see Fig. S34). (The scanning electron micrograph of the Early Medieval Egyptian blue paint layer in Fig. 3 of Ref.^17^ shows such quartz grains intergrown with cuprorivaite.) Note that the present study revealed a significantly higher average crystallinity of ancient cuprorivaite compared to the modern counterpart (Kremer Pigmente), evidencing differences within their specific synthesis parameters (see Fig. S33; the Raman spectra of the Early Medieval cuprorivaite discussed in Ref.^17^ match the range of band widths and shifts of the Roman Imperial analogue). The interpretation of the downshift of the 1087 cm^−1^ band as consequence of weakening of the layer structure was further corroborated by the analysis of finely ground modern cuprorivaite; mechanically damaging the sheet structure using a mortar lead to a further spread of the Raman data towards lower wavenumbers with a minimum of 1072 cm^−1^, whereas the band widths (representing the overall crystallinity resulting from the process conditions) remained in the same range (see Figs. S32b, S33).

#### By-products of the synthesis

Wollastonite CaSiO_3_ rarely occurs as an intrinsic by-product of the Egyptian blue manufacture (see Figs. S35, S36). The pyroxenoid might be formed at the calcite–quartz interface in consequence of (local) excess of calcium, seen the temperature range of 850 °C to 1000 °C derived from laboratory experiments as appropriate for the formation of cuprorivaite^9,11–16,37^. (During ceramic firing wollastonite appears already at 800 °C in very low concentrations as reaction rim between carbonates and silicates^55–60^). Likewise, the sporadic detection of cristobalite in the pigment balls under study arises from excessively high synthesis temperatures or at least locally high concentration of alkali flux. An alternative hypothesis would imply the presence of this high-temperature polymorph of SiO_2_ as subordinate mineral in the processed quartz sand.

#### Green glass phase

In the course of the rediscovery of Egyptian blue at the turn of the century and the establishment of the analytical chemistry during the nineteenth century, numerous laboratory experiments were performed to determine the optimal process conditions and the spectrum of possible reaction products to be encountered in the blue pigment^1,14,61^. Until today, the extensive results are reflected in contradictory interpretations in particular with regard to the formation of an amorphous phase. Ferdinand Fouqué, for example, observed the decomposition of cuprorivaite in the temperature range above bright red into wollastonite, dendritic crystals of copper oxide and a light green glass phase; when white hot, wollastonite decomposed, leaving only the aventurine-green glass, embedding minute crystals of copper oxide^62^. Likewise, Gerhard Bayer and Hans-Georg Wiedemann as well as Detlef Ullrich reported the breakdown of cuprorivaite above 1050 °C, leading to the coexistence of copper oxides, silica and wollastonite^16,43,63^. Pierluigi Bianchetti et al. and Ioanna Kakoulli, by contrast, depicted the presence of wollastonite, copper oxides and a light blue or pale green glass at temperature values significantly below the decomposition of cuprorivaite^13,64^, whereas Arthur Laurie et al. circumstantiate the formation of an olive-green glass phase at 800 °C, thus already prior to the pursued crystallisation of the blue mineral “somewhere about 830°”; this amorphous phase again predominates when the synthesis temperature is raised above 900 °C^65^.

We discerned a green amorphous phase on the Roman Imperial Egyptian blue balls unearthed in the remains of the ancient cities of Aventicum and Augusta Raurica (Fig. 2), which might be associated with small-scale compositional inhomogeneities, i.e. a locally high flux concentration—for example, due to transport of soluble salts towards the surface during the drying of the balls^3^—might have given rise to a liquid phase consuming the quartz grains, thus facilitating the diffusion of the chromophoric Cu^2+^ ions into the liquid before the onset of solid-state reactions, which in turn lead to the crystallisation of cuprorivaite. Possibly this green glass is concordant to green particles observed by Ariadne Kostomitsopoulou Marketou et al. in pigment balls from a first century B.C. workshop of the Greek island of Kos and specified as a green Raman-silent Cu–Si glass resulting from an interrupted secondary treatment step^66^. Seen the case under discussion here, this spectroscopic interpretation might be explained by the broad Raman bands attributable to the amorphous phase exhibiting intensities around one order of magnitude lower than the Raman spectra gathered from cuprorivaite and thus difficult to identify within complex mixtures (see Fig. 4, Fig. S37).

The signature of the green glass phase (see Figs. S38–S40) resembles the one of ancient alkaline glasses (and enamels)^34,67–70^: broad and superimposed bands in a low-wavenumber range from approx. 280 cm^−1^ to 720 cm^−1^ assigned to bending vibrations of differently interconnected SiO_4_^4−^ tetrahedra and an according stretch-vibrational high-wavenumber range from approx. 850 cm^−1^ to 1200 cm^−1^. Different interpretations exist for the mid-range region, which in the spectra of some (ancient) glasses contains weak bands. While a doublet with a significant intensity at > 800 cm^−1^ observed in some silicate glasses with high SiO_2_ content is hypothesised as due to a symmetric motion of Si against its cage of O atoms^71,72^, we see an obvious analogy of the mid-range bands at around 785 cm^−1^ (and no features at > 800 cm^−1^) to a peak monitored by Justyna Sułowska et al. to raise in intensity, when increasing the amount of Cu^2+^ added to silicate glasses^73^. A clearly discernible peak occurs in the spectrum of a glass with the major elements Si, Ca, Mg and Cu in the molar ratio of 4:1.4:1.2:1.8, thus, not fundamentally but significantly diverging from the Si:Ca:Cu = 4:1:1 stoichiometry of cuprorivaite. We interpret these mid-range bands as bending vibrations of four-membered silicate rings coordinated with Cu^2+^ (see Figs. S38–S40; vibrational features in the same wavenumber range of crystalline forms of such ring structures are described in Refs.^53,74^). This band allows a clear distinction from other glass compositions, so for example from the copper-free and thus colourless amorphous phase formed upon heating pure modern cuprorivaite up to 1300 °C, whose Raman spectrum misses bands in the mid-range region (see Figs. S41–S43; the result did not significantly change when thermally decomposing modern Egyptian blue (Kremer Pigmente) mixed with sodium hydrogencarbonate as flux, see Figs. S45–S48).

Due to the current lack of reference Raman data in the scientific literature, it is not feasible to find the equivalent of the green amorphous phase spotted on the Roman Imperial pigment balls under study in the copper-bearing glass responsible for the distinguishing green to turquoise, at times even blue hue of the likewise artificial pigment Egyptian green, characterised by the coincident presence of wollastonite and the high-temperature SiO_2_ polymorphs tridymite and cristobalite. Its manufacture from a proportionally modified mixture of the same compounds used for the synthesis of Egyptian blue in a higher temperature range and its art technological application seem to be confined almost entirely to the Egyptian territory, primarily to the New Kingdom era^11–13,75^. However, fragments of globular crucibles covered with residues of green colour witness the parallel production of Egyptian blue (in cylindrical crucibles) and Egyptian green at Cumae (Gulf of Pozzuoli, Campania, Southern Italy) at least in the course of the first century B.C.; according to Celestino Grifa et al. newly formed minerals, particularly a sodalite-nosean feldspathoid, confirm the exposure of the ceramic objects to temperatures above 1050 °C^6^.

### Contaminations by adherent soil minerals

Fluvioglaciale sediments and uncemented rocks in the Augusta Raurica as well as Aventicum area embrace, amongst others, fragments of granite, quartzite and schist^76,77^. Strong autofluorescence typical for soil organic matter, hampering analyses by Raman microspectroscopy, and the Raman signature of humic substances^78^ provided evidence for the identification of some of the traceable minerals as inorganic components of soil, thus as contaminations of the sample material in consequence of abandonment of the ancient structures and not as natural impurities of the raw material blend for the manufacture of the Egyptian blue balls (Fig. S51).

The phyllosilicate stilpnomelane K(Fe,Mg,Al)_8_(Si,Al)_12_(O,OH)_27_·2H_2_O (Fig. S49) occurs in a large range of compositions as a common mineral of low-grade metamorphism along with chlorite, muscovite and albite in greenschists, furthermore in glaucophane-lawsonite facies (blueschists) and ironstones. On heating, it first loses interlayer water molecules and above about 450 °C Fe^2+^ is progressively oxidised and equivalent structural OH is lost^79^, which makes the attribution to inorganic soil components plausible.

In addition, the ubiquitous sheet silicate muscovite KAl_2_(Si_3_Al)O_10_(OH,F)_2_ was detectable by means of Raman microspectroscopy (Fig. 3, Figs. S50, S51). White mica dehydroxylation, accompanied by delamination, occurs over a considerable temperature interval; the platy structure is decomposed only on firing to temperatures above 1000 °C^55–58,80–84^. As dilation of the crystal lattice and delamination should affect the Raman spectra (see Fig. S52), we conclude that muscovite—possibly just as biotite subordinate mineral of the processed quartz sand^21–26^—was not involved in the synthesis of Egyptian blue and/or its presence on the surface of the studied pigment balls left in earth for centuries is due to contact with soil and cautious cleaning after excavation.

The same applies to gypsum CaSO_4_·2H_2_O (Fig. S53) seen its conversion or dehydration, respectively, into bassanite (hemihydrate) CaSO_4_∙½H_2_O and anhydrite III (soluble anhydrite)^85^ during the Raman measurements through the influence of colocalised organic chromophores in the form of humic substances (Fig. S54); the thermal transformation of calcium sulphate dihydrate is thus triggered by local heating-up comparable to well-known laser-induced alterations of coloured sulphide and oxysalt minerals during Raman experiments^86–88^.

## Conclusions: evidences for the provenance of the raw materials and therewith of the Egyptian blue pigment balls

The study sheds light on the trace compounds characterising Egyptian blue balls and mural paintings, excavated in the archaeological remains of the cities Aventicum and Augusta Raurica, dated via stratigraphically associated finds to the middle of the first century A.D., the beginning of the second century A.D. as well as the first half of the third century A.D. With regard to the question of whether the Roman Imperial pigment is imported from the northern Phlegrean Fields in Campania (Southern Italy) or manufactured on site in Switzerland, the accessories attributable to the quartz sand used embody relevant indications, in particular the clinopyroxenes aegirine NaFeSi_2_O_6_ and augite and the seldom barium-rich alkali feldspar celsian BaAl_2_Si_2_O_8_. As in the case of the Early Medieval Egyptian blue applied in the course of the fifth or sixth century A.D. in St. Peter above Gratsch (South Tyrol, Northern Italy)^17^, a sulphidic copper ore (i.e. chalcocite and chalcopyrite accompanied by different sulphides, selenides, arsenates, chromates and members of the spinel group) necessarily roasted to yield copper oxide, was employed as copper source. Likewise, the addition of an alkaline flux in the form of soda-rich or mixed-alkaline plant ash was reinforced due to the detection of mainly sulphate and phosphate salts of sodium and potassium as well as magnesium and calcium. Such corresponding trace constituents in Roman Imperial and Early Medieval Egyptian blue provide sound scientific evidence of a continuous production and trade monopoly in the Gulf of Pozzuoli surviving from the first centuries A.D. up to the politically turbulent period after the fall of the Western Roman Empire, this in line with statements of the antique Roman writers Vitruvius^3^ and Pliny the Elder^4^ and recent archaeological finds in the cities of Cumae and Liternum^6–8^.

Beyond that, Raman microspectroscopy provided valuable insights into the thermal history of the ancient artificial blue pigment: Raman spectra of cuprorivaite exhibiting gradual peak shifts and changes of band width revealed crystal lattice disorder due to insufficient reaction time, this alongside with remnant quartz grains intergrown with cuprorivaite (also compare scanning electron micrograph of a cross-sectional sample of the Early Medieval pictorial layer in Ref.^17^). Intense comminution of the raw materials facilitated solid-state reactions during the manufacture of the Roman Imperial Egyptian blue; melting most likely played a negligible role, since a copper-bearing green glass phase could be observed only locally restricted on the surface as a result of the abundant availability of fluxing agents. In conclusion, Raman microspectroscopically monitored syntheses are needed for the evaluation of these hypotheses of formation conditions of the observed crystalline as well as amorphous constituents, and of the effect of parameters such as reaction time, temperature^89^, and annealing^90^ on the observed disorder in the cuprorivaite structure.

## Supplementary Information

Supplementary Information.

## Data Availability

The datasets generated during the current study are available from the corresponding author on reasonable request.
