# Supplementary material for: Raman focal point on Roman Egyptian blue elucidates disordered cuprorivaite, green glass phase and trace compounds
Source: Sci Rep. 2022 Sep 16;12:15596. doi: 10.1038/s41598-022-19923-w (PMC9481618; doi:10.1038/s41598-022-19923-w)
Supplement: Supplementary file 1 — Supplementary Information. [file 41598_2022_19923_MOESM1_ESM.pdf]

Supplementary Information for

Raman focal point on Roman Egyptian blue elucidates disordered cuprorivaite, green glass phase and trace compounds

Petra Dariz[1], and Thomas Schmid[2,*]

[1] Bern University of the Arts, 3027 Bern, Switzerland

[2] Bundesanstalt für Materialforschung und -prüfung (BAM), 12489 Berlin, Germany

*thomas.schmid@bam.de or thomas@schmid.eu.com

Samples

Table S1. Roman Imperial and Early Medieval Egyptian blue samples analysed by Raman microspectroscopic imaging in the present and predecessor study [17].

Site	Sample	Approximate dating	Photo
Aventicum (Canton of Vaud, Switzerland)	Pigment ball 'forum'	second half of 1st century/beginning of 2nd century A.D.	Fig. 1 (top left)
	Pigment ball 'ins15'	second half of 1st century/beginning of 2nd century A.D.	Fig. 1 (top right)
Augusta Raurica (Canton of Basel-Country, Switzerand)	Pigment ball	second/third quarter of 1st century A.D.	Fig. 1 (bottom left)
	Mural fragment	first half of 3rd century A.D.	Fig. 1 (bottom right)
St. Peter above Gratsch (South Tyrol, Italy) [17]	Mural fragment	5th/6th century A.D.	Fig. 2 in Ref. [17]

Remarks on the assignments of Raman spectra to minerals

Figs. S3–S28, S35–S36, S46, S49–S50, and S53–S54 compare selected Raman spectra of Roman Imperial (red) and Early Medieval Egyptian blue (green) [17] with reference data from the RRUFF spectral library (blue) [19] for validating their assignments (Xnnnnnn and Rnnnnnn are RRUFF ID numbers). The acronyms 'forum' and 'ins15' (insulae 15) refer to different excavation sites and campaigns in Aventicum.

Light microscopy

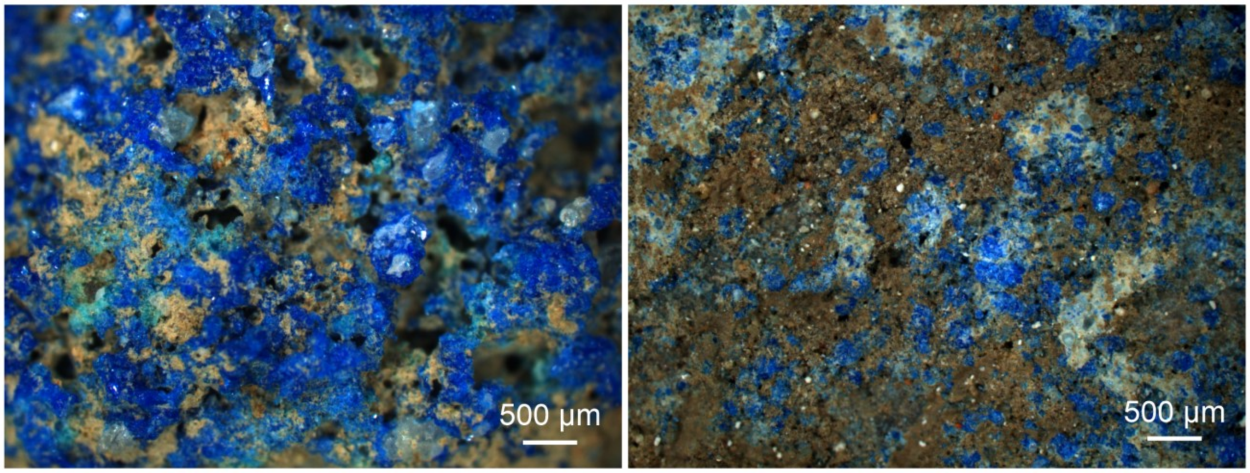

Figure S1. Darkfield light microscopy images of the pigment ball unearthed in Aventicum (left; forum) and the pictorial layer of the mural fragment from Augusta Raurica (right). Brown particles present in both images are due to adherent soil from the excavation sites. See Fig. 2 in the main text for further micrographs.

Raman microspectroscopic imaging

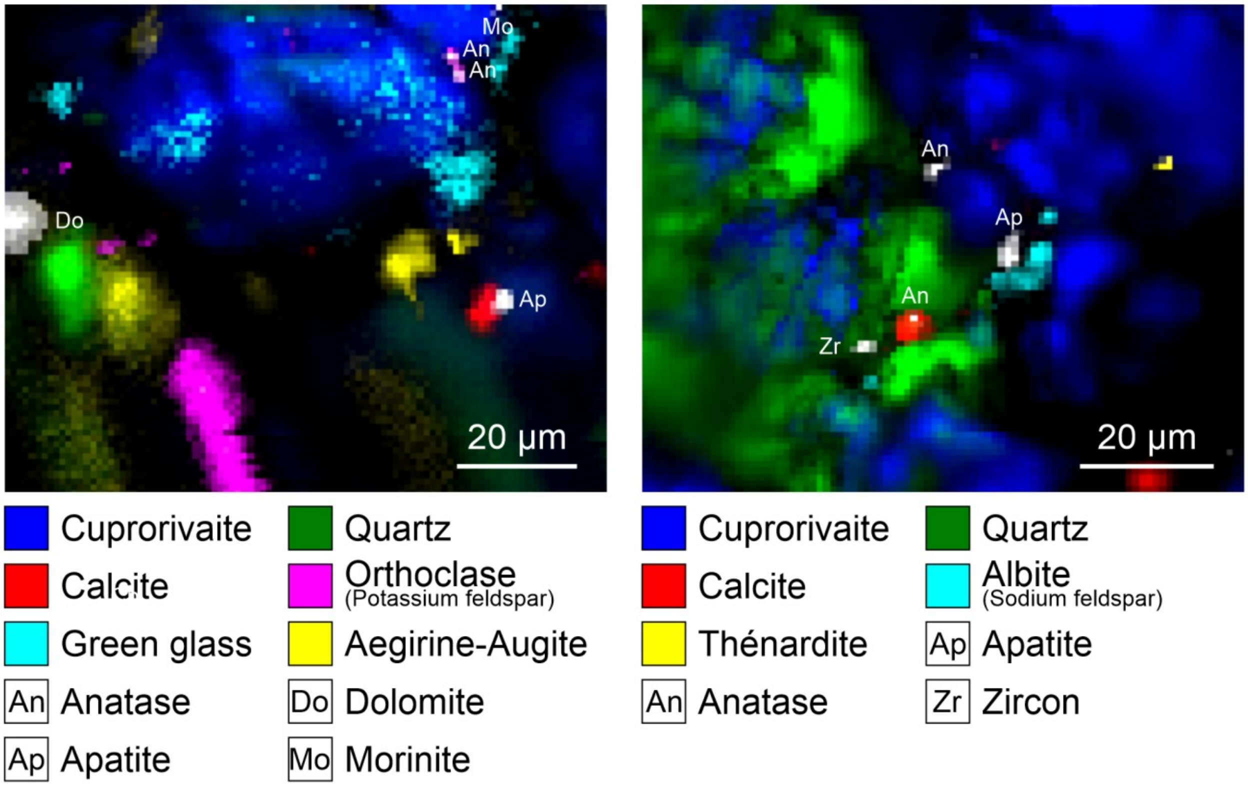

Figure S2. Selected Raman maps of the pigment balls from Aventicum, i.a. comprising trace compounds of the quartz sand (left; forum) and thénardite from the alkali flux (right; insulae 15). See Fig. 3 in the main text for further Raman microspectroscopic images.

Mineralogy of the quartz sand

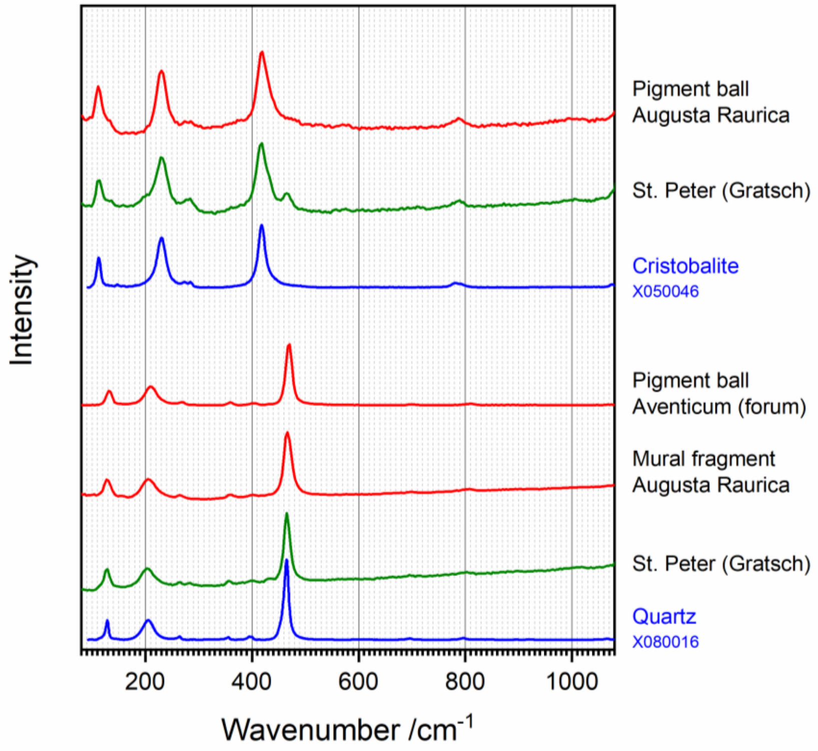

Figure S3. Quartz SiO_2 and the high-temperature SiO_2 polymorph cristobalite.

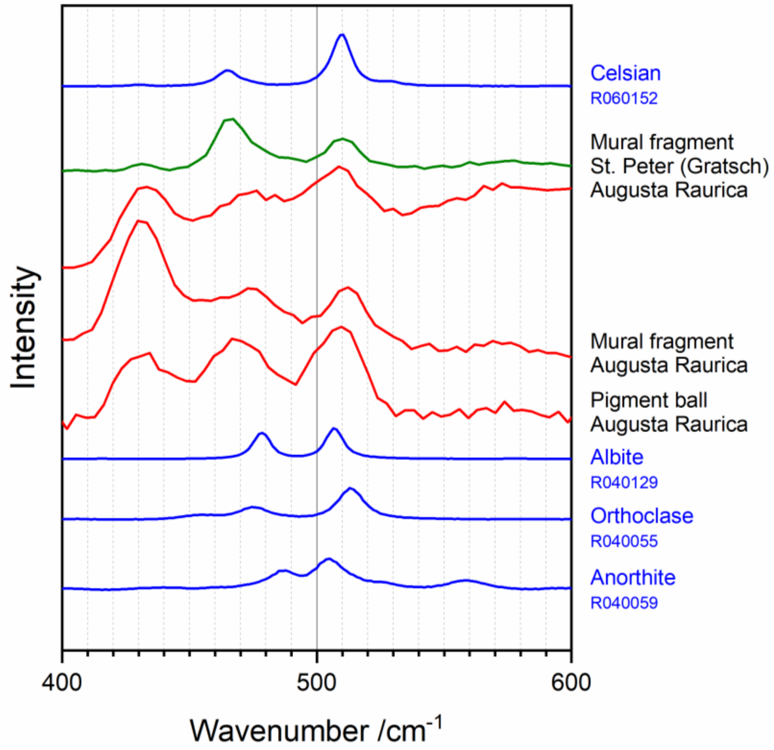

Figure S4: Feldspars found in Egyptian blue compared with reference data of anorthite $CaAl_2Si_2O_8$, orthoclase $KAlSi_3O_8$, albite $NaAlSi_3O_8$, and celsian $BaAl_2Si_2O_8$.

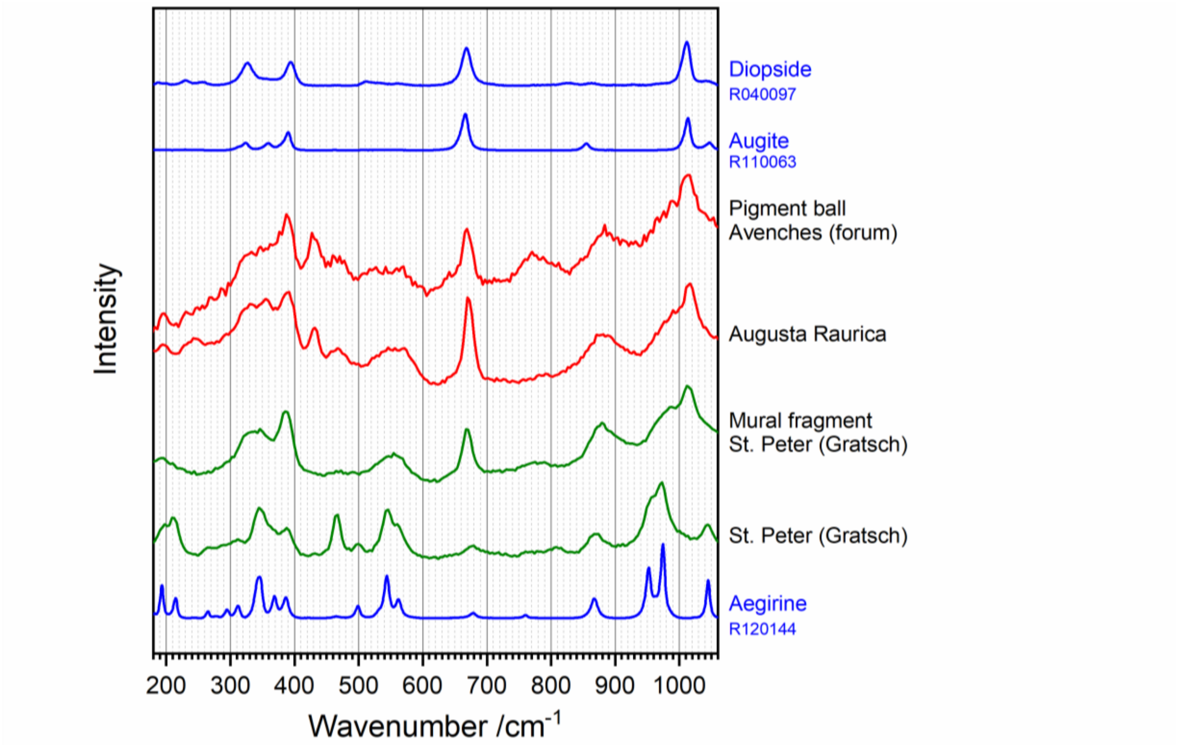

Figure S5: Aegirine $NaFeSi_2O_6$, augite $(Ca,Mg,Fe)_2Si_2O_6$ and/or diopside $CaMgSi_2O_6$ (discrimination not possible based on the available reference data), and colocalised aegirine-augite.

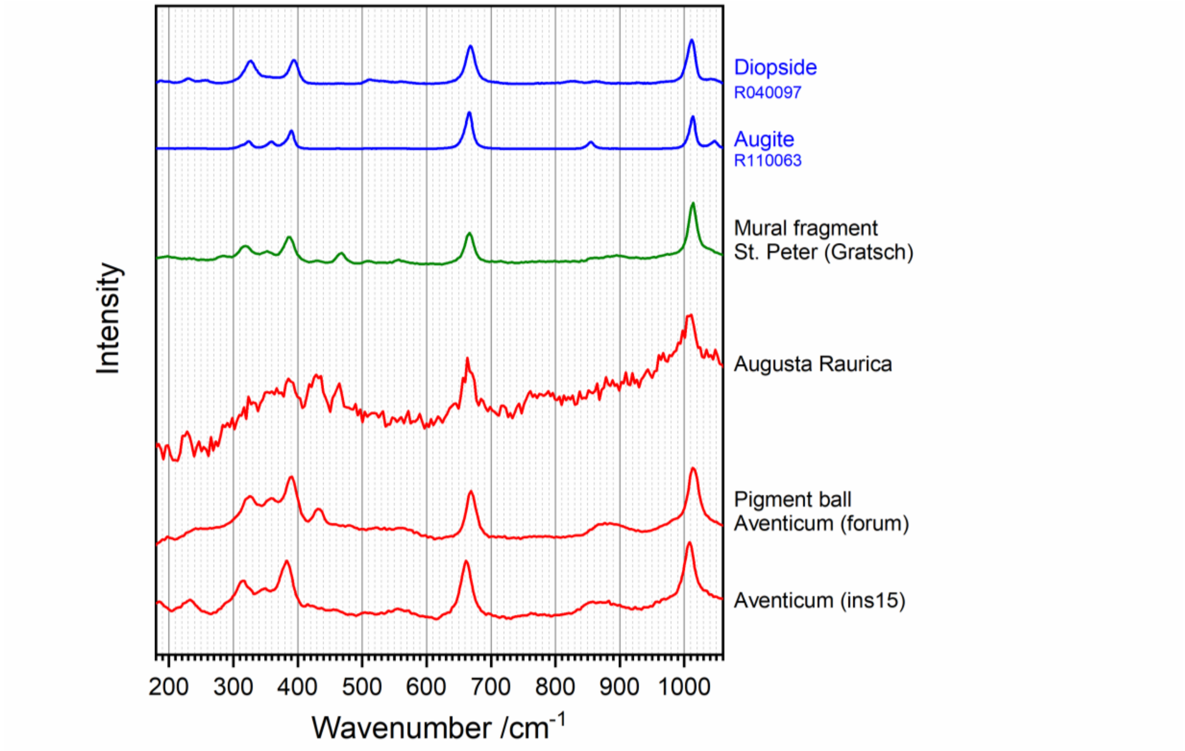

Figure S6: Augite $(Ca,Mg,Fe)_2Si_2O_6$ and/or diopside $CaMgSi_2O_6$, which cannot be distinguished based on the available reference data.

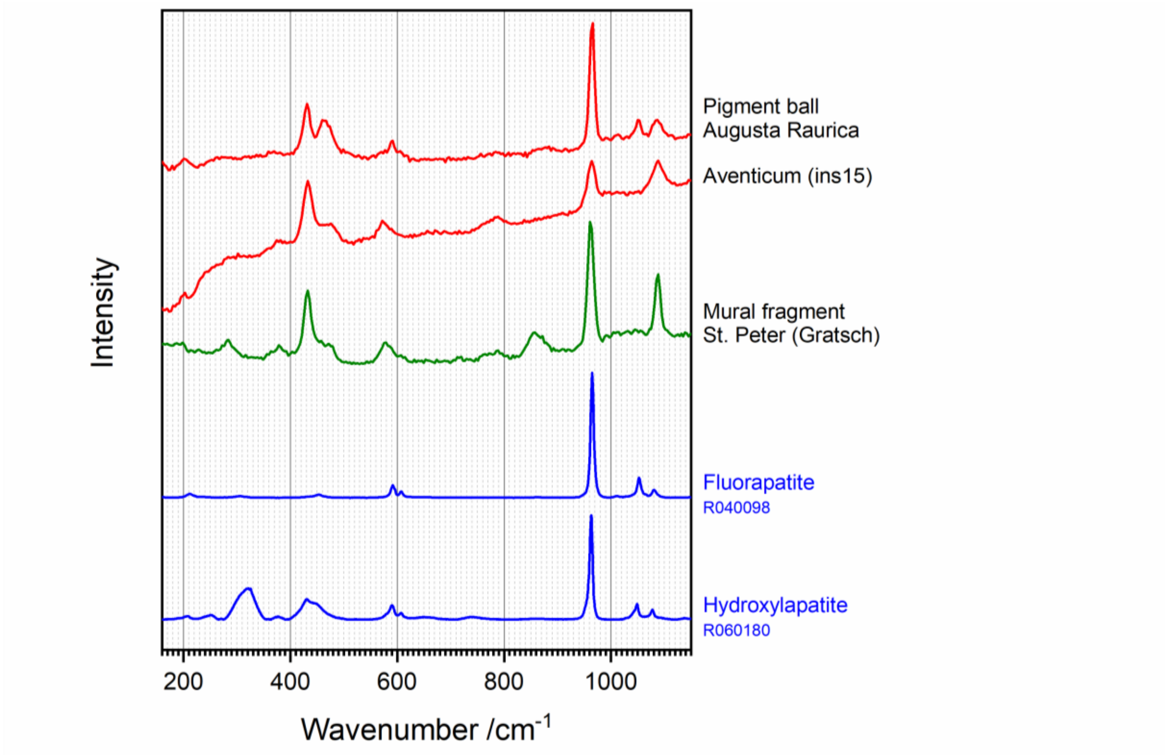

Figure S7: Apatite $Ca_5(PO_4)_3(OH,F)$.

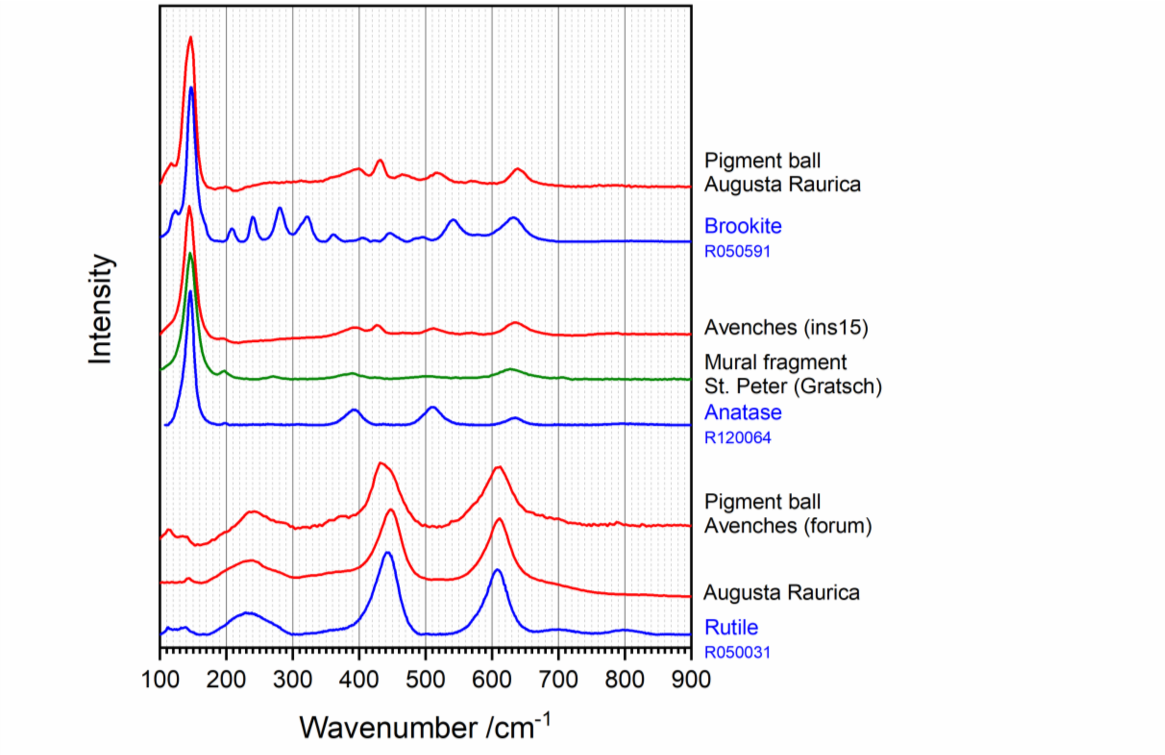

Figure S8: Rutile and the other TiO_2 polymorphs anatase and brookite.

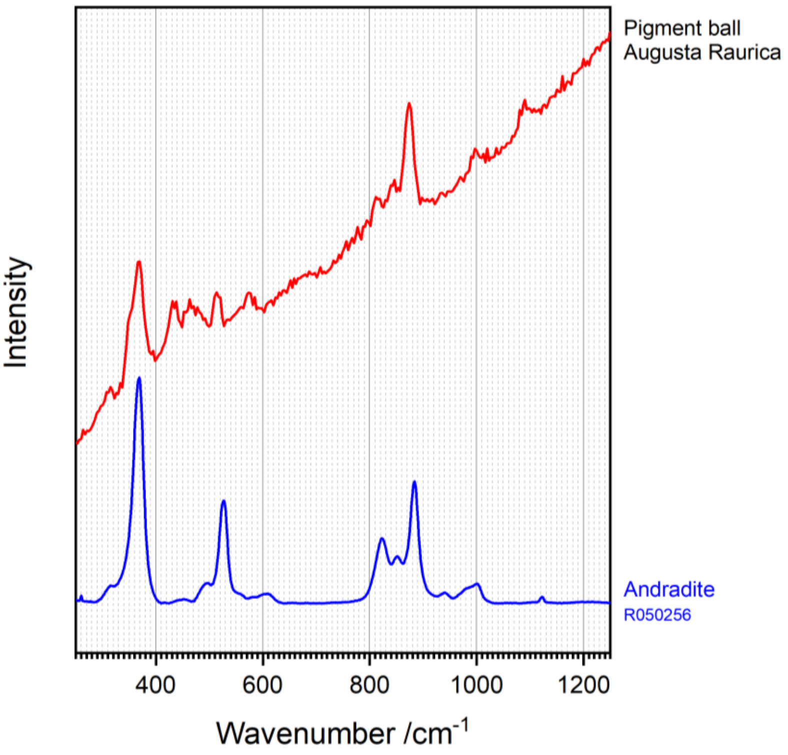

Figure S9: The garnet andradite $Ca_3Fe_2Si_3O_{12}$.

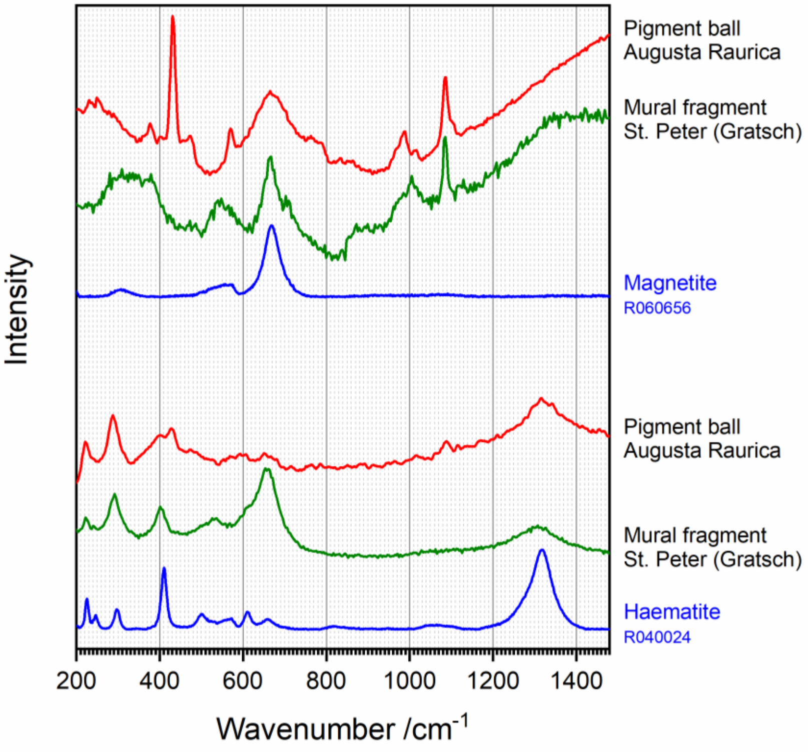

Figure S10: The iron oxides haematite α-Fe_2O_3 and magnetite Fe_3O_4 (spinel group).

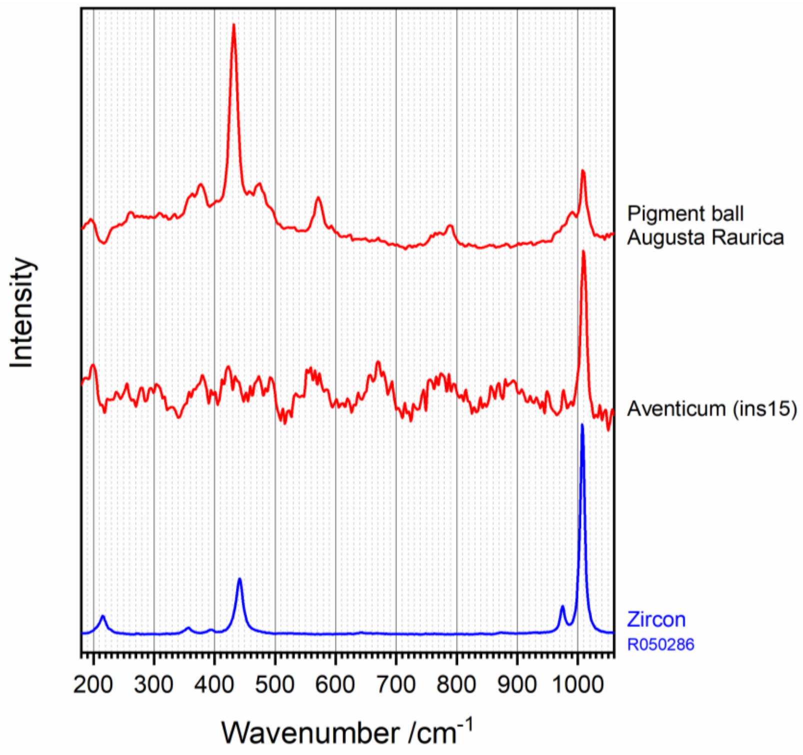

Figure S11: Zircon $ZrSiO_4$.

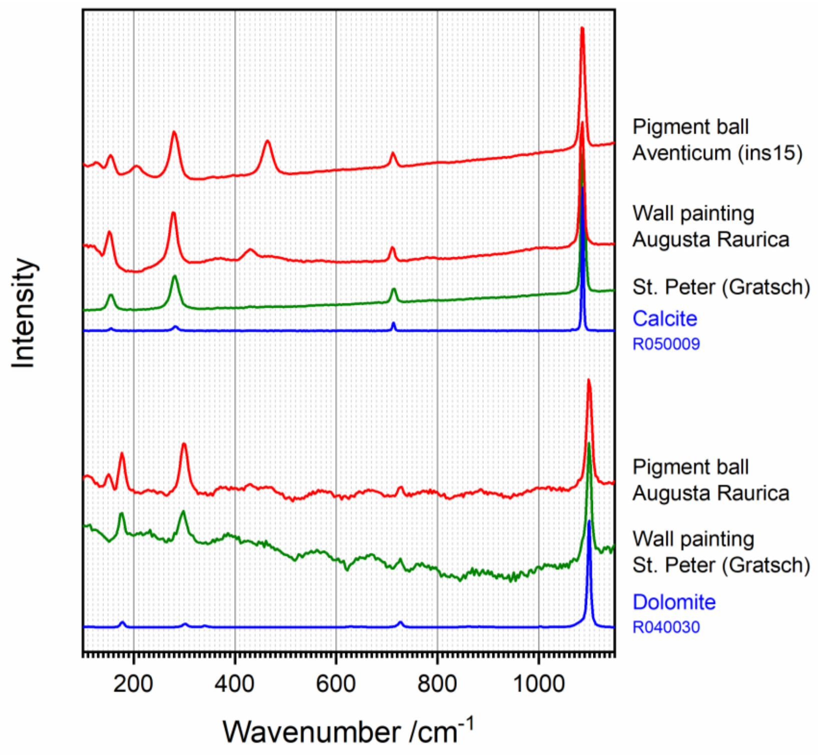

Figure S12: The carbonates calcite $CaCO_3$ and dolomite $CaMg(CO_3)_2$.

Constituents and accessory minerals of the copper ore

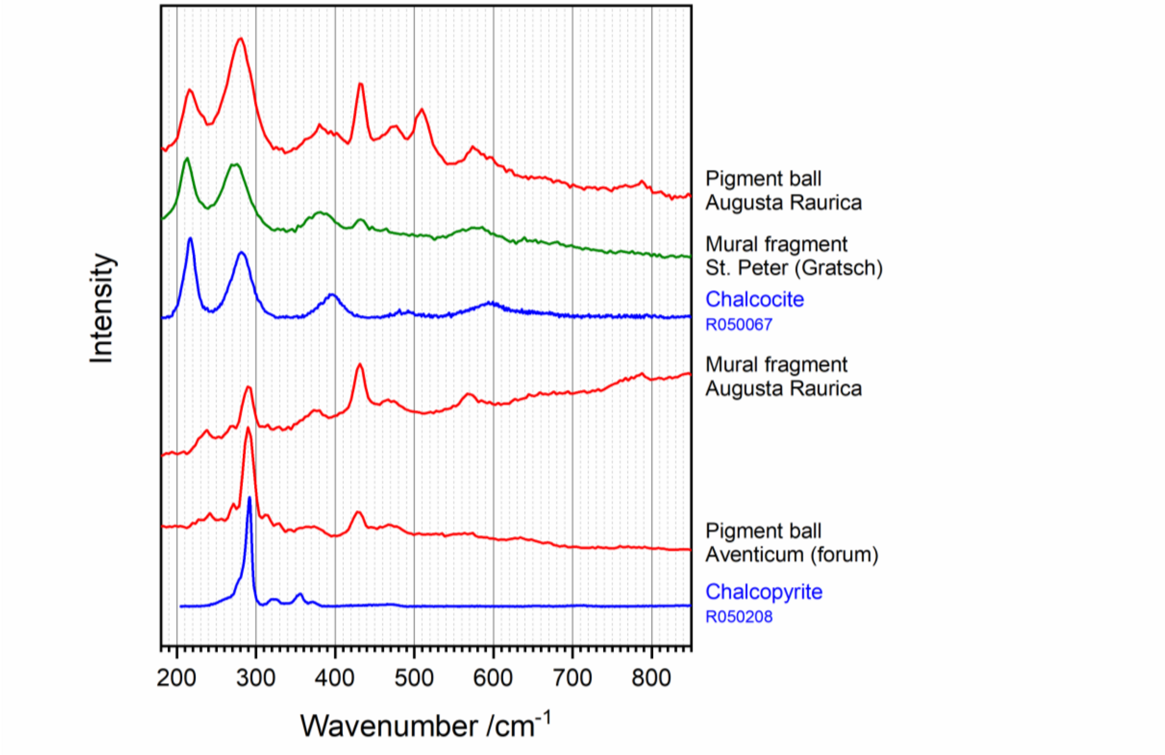

Figure S13: Major constituents of a sulphidic copper ore: chalcopyrite $CuFeS_2$ and chalcocite Cu_2S.

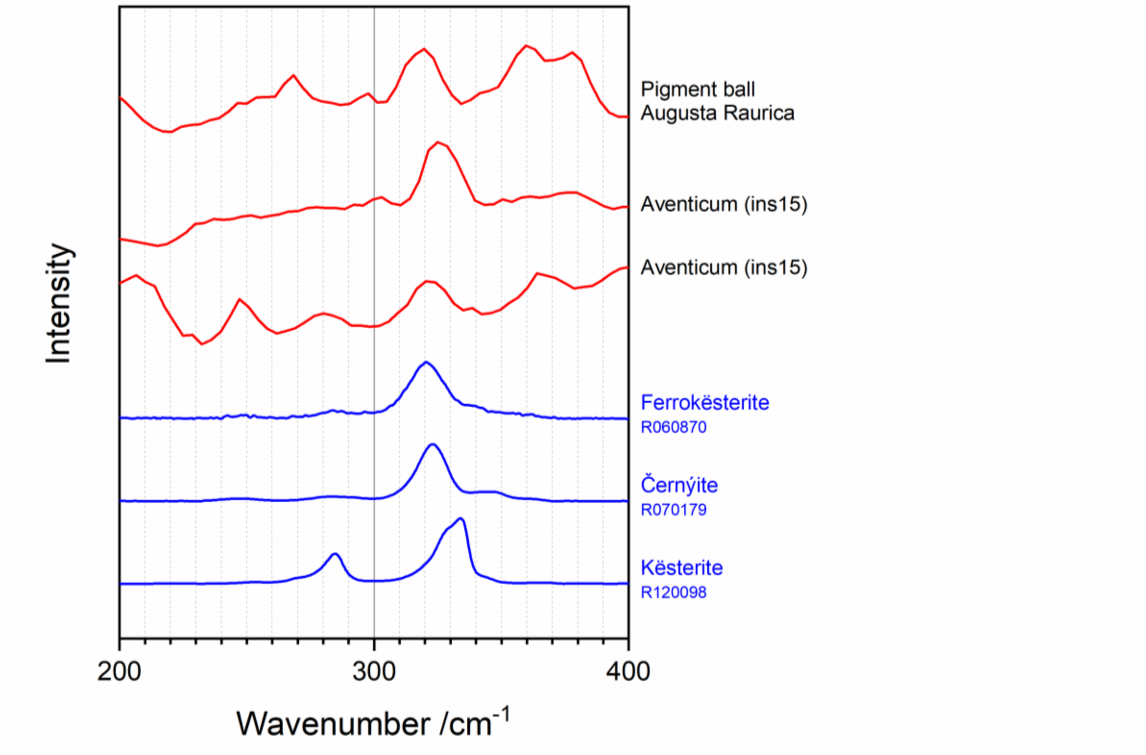

Figure S14: Kësterite group minerals: Kësterite $Cu_2(Zn,Fe)SnS_4$, Černýite Cu_2CdSnS_4, and Ferrokësterite Cu_2FeSnS_4.

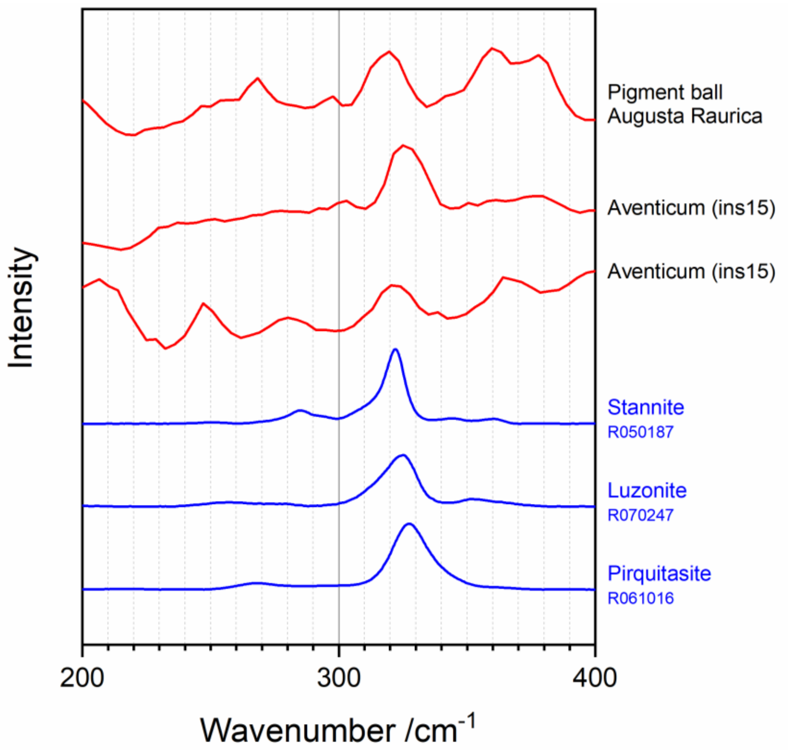

Figure S15: Stannite group minerals: Stannite Cu_2FeSnS_4, Luzonite Cu_3AsS_4, and Pirquitasite Ag_2ZnSnS_4.

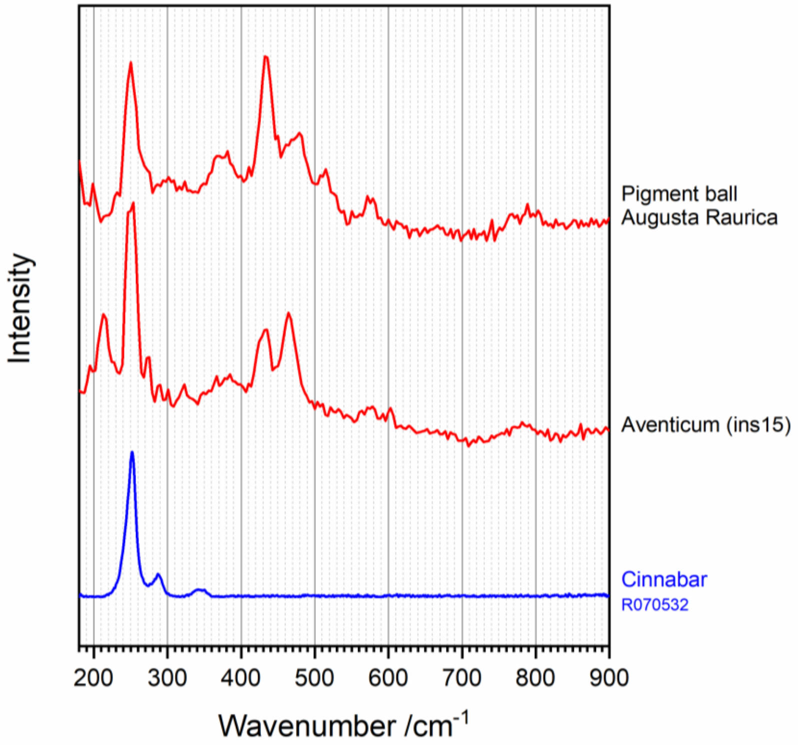

Figure S16: Cinnabar HgS.

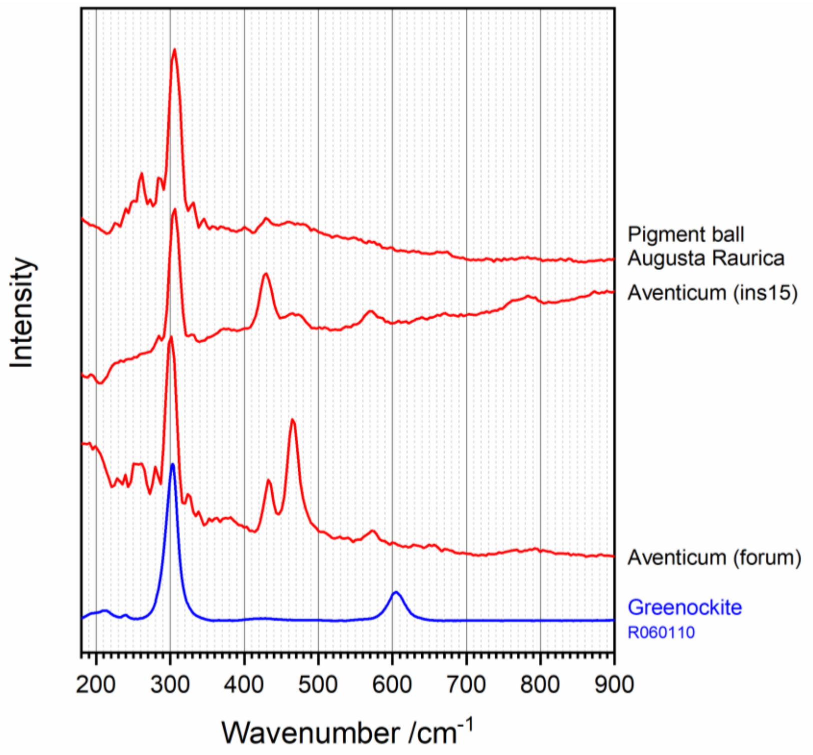

Figure S17: Greenockite CdS.

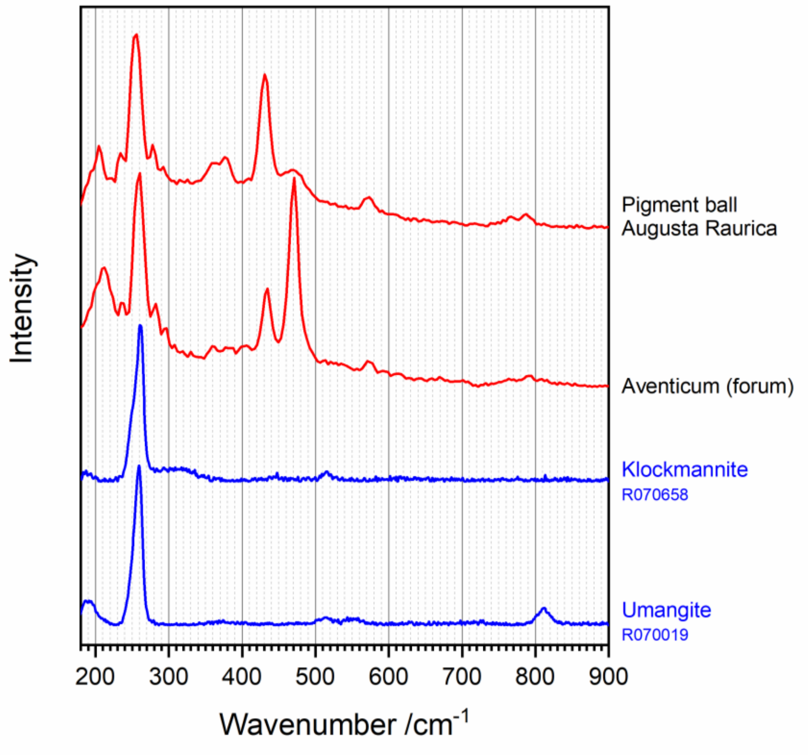

Figure S18: Klockmannite CuSe or umangite Cu_3Se_2.

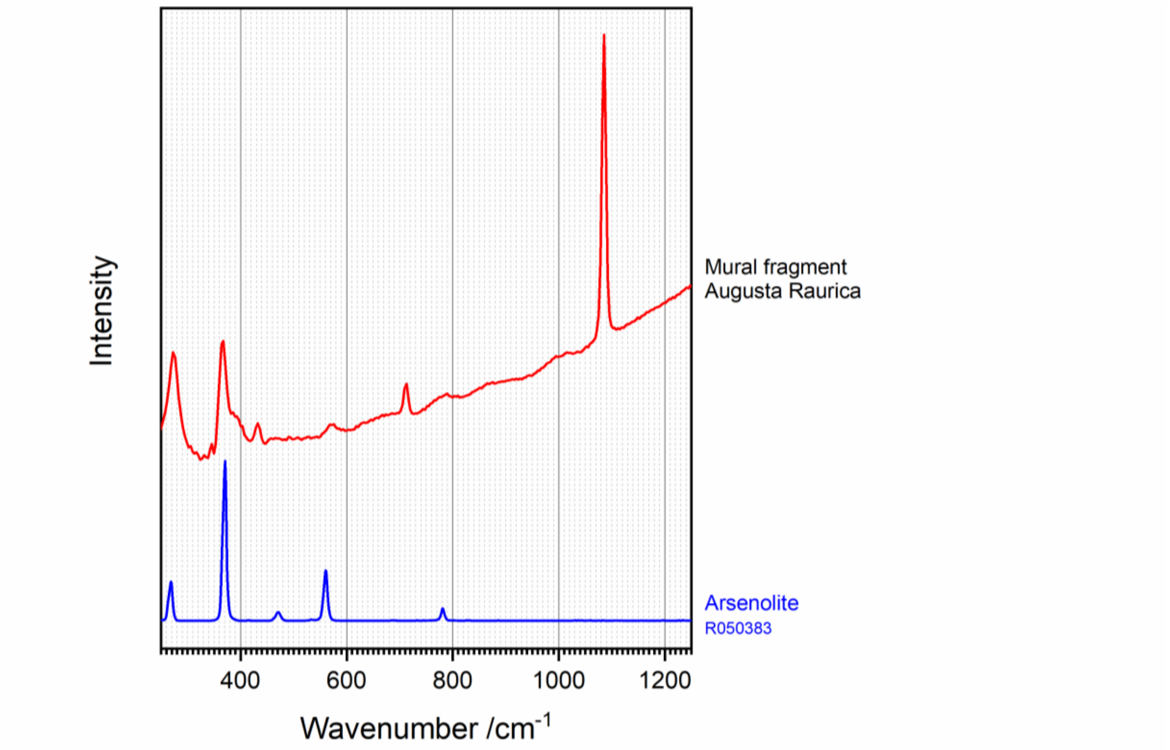

Figure S19: Arsenolite As_2O_3.

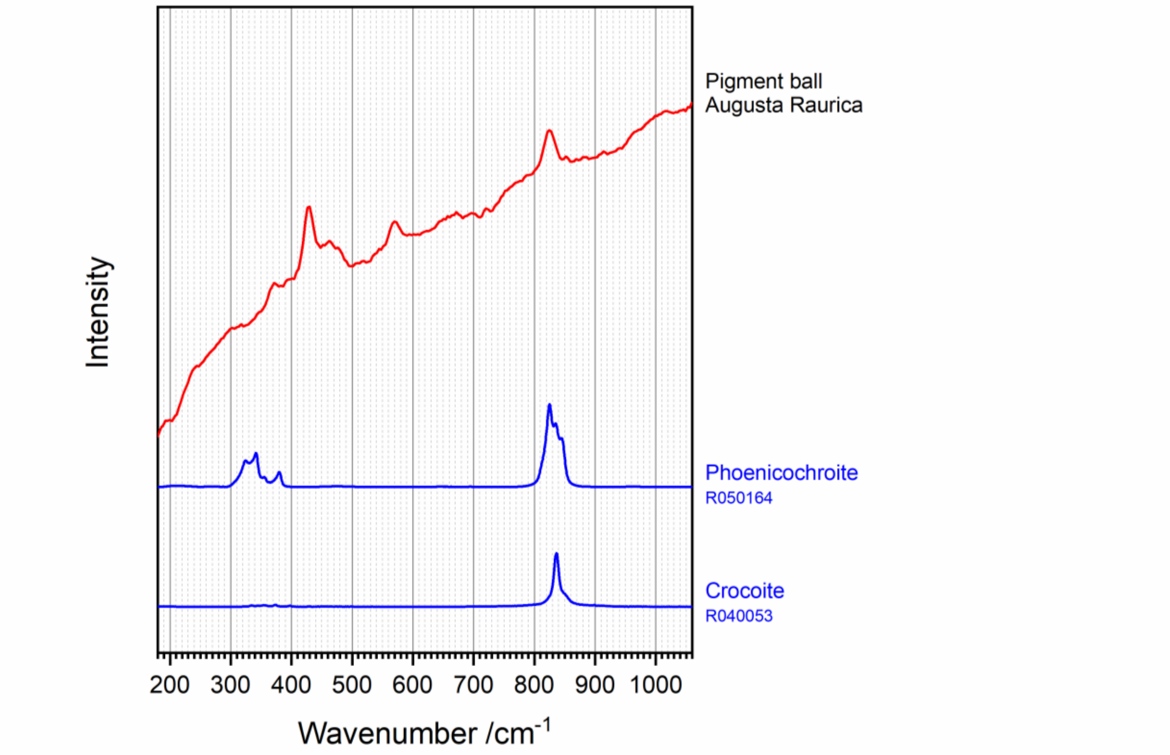

Figure S20: Phoenicochroite $Pb_2(CrO_4)O$ or crocoite $PbCrO_4$.

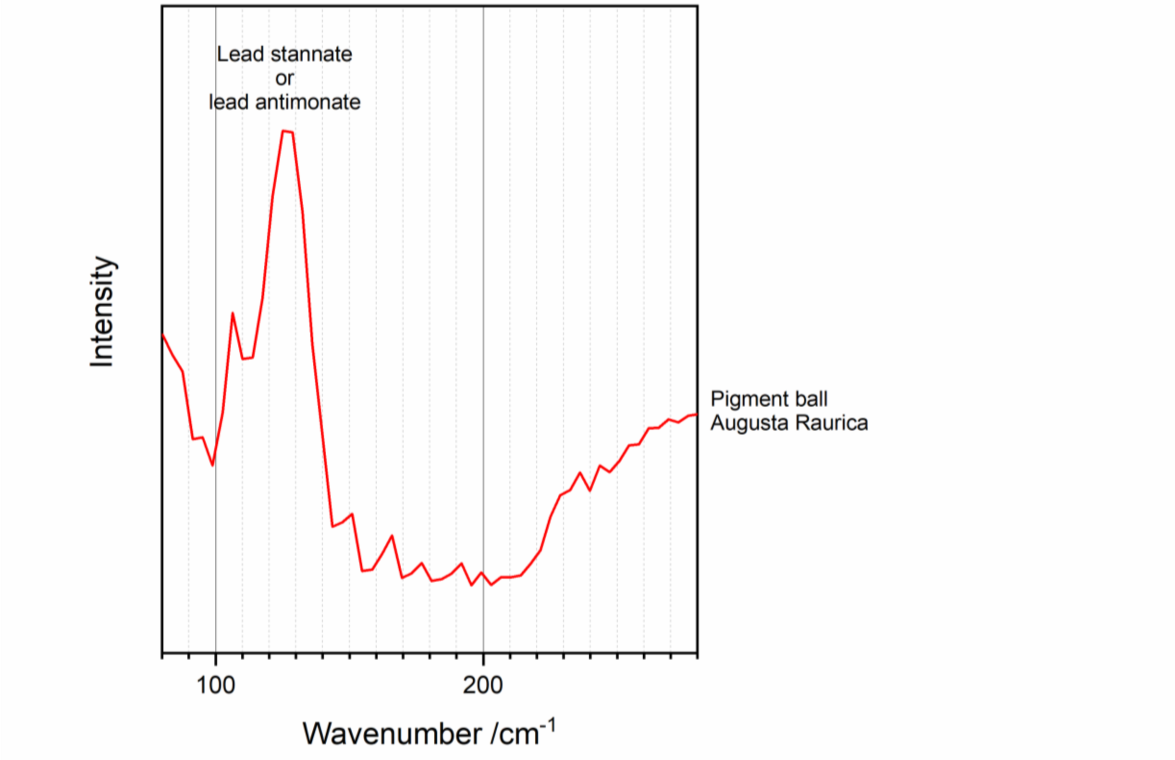

Figure S21: Lead stannate (Lead tin yellow I) Pb_2SnO_4 or lead antimonate (Napes yellow) $Pb_2Sb_2O_7$ [S1].

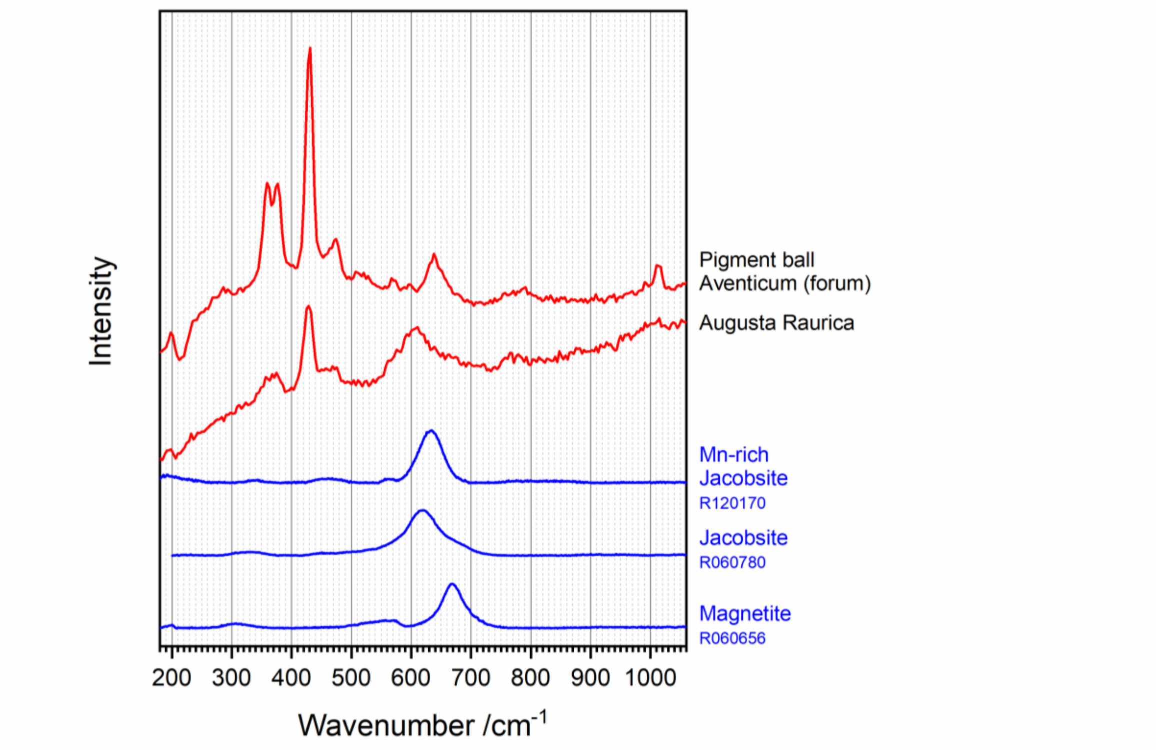

Figure S22: The spinel group minerals magnetite Fe_3O_4 and jacobsite $MnFe_2O_4$, and members of related solid solution series detected in Roman Imperial Egyptian blue.

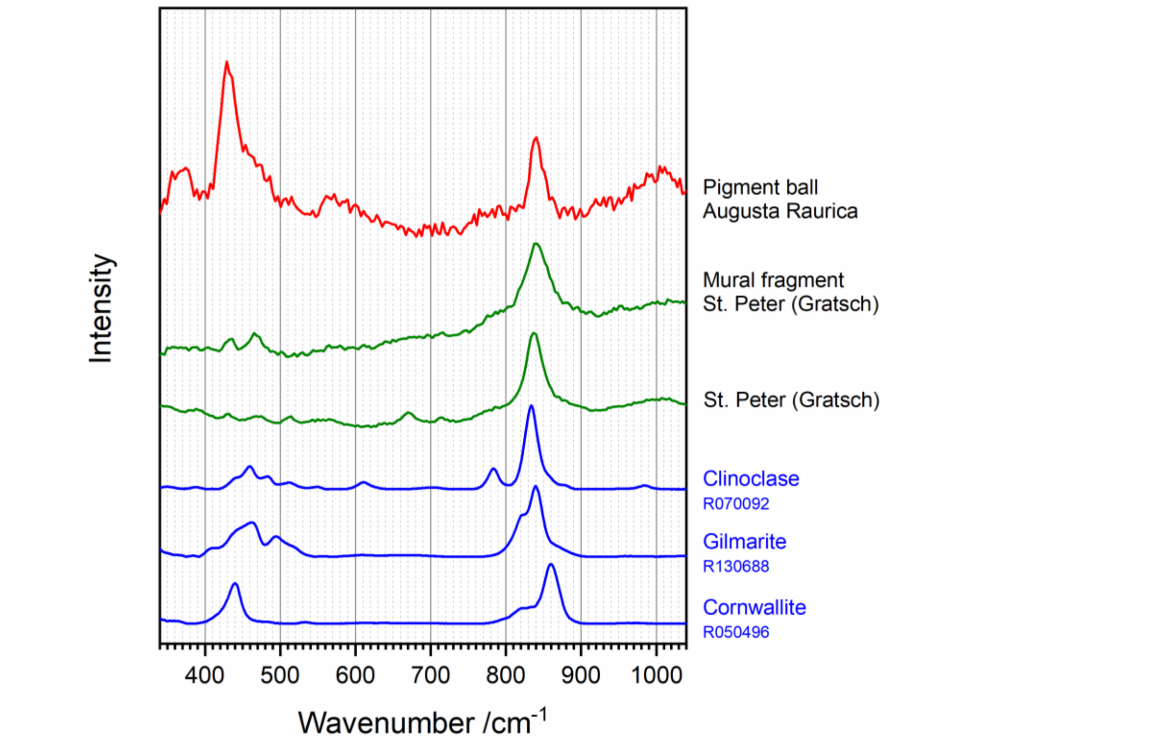

Figure S23: Clinoclase $Cu_3(AsO_4)(OH)_3$, gilmarite $Cu_3AsO_4(OH)_3$, and cornwallite $Cu_5(AsO_4)_2(OH)_4$ representing basic copper arsenates.

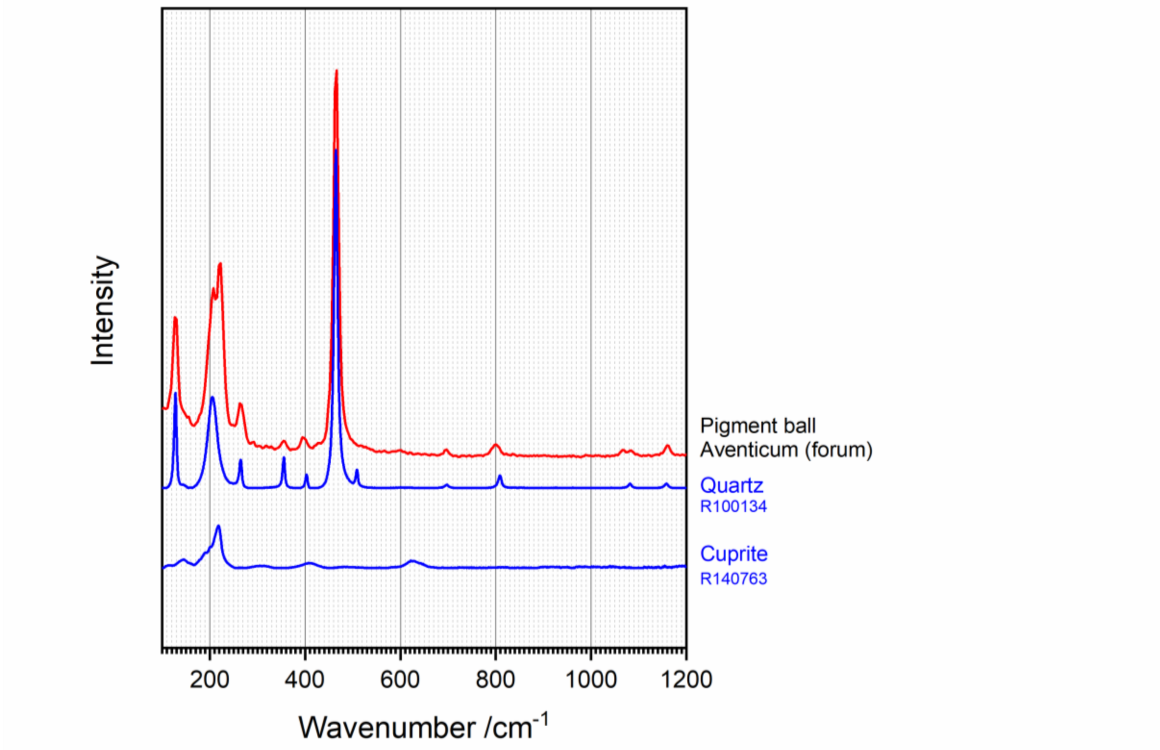

Figure S24: Cuprite Cu_2O.

Type of alkali flux

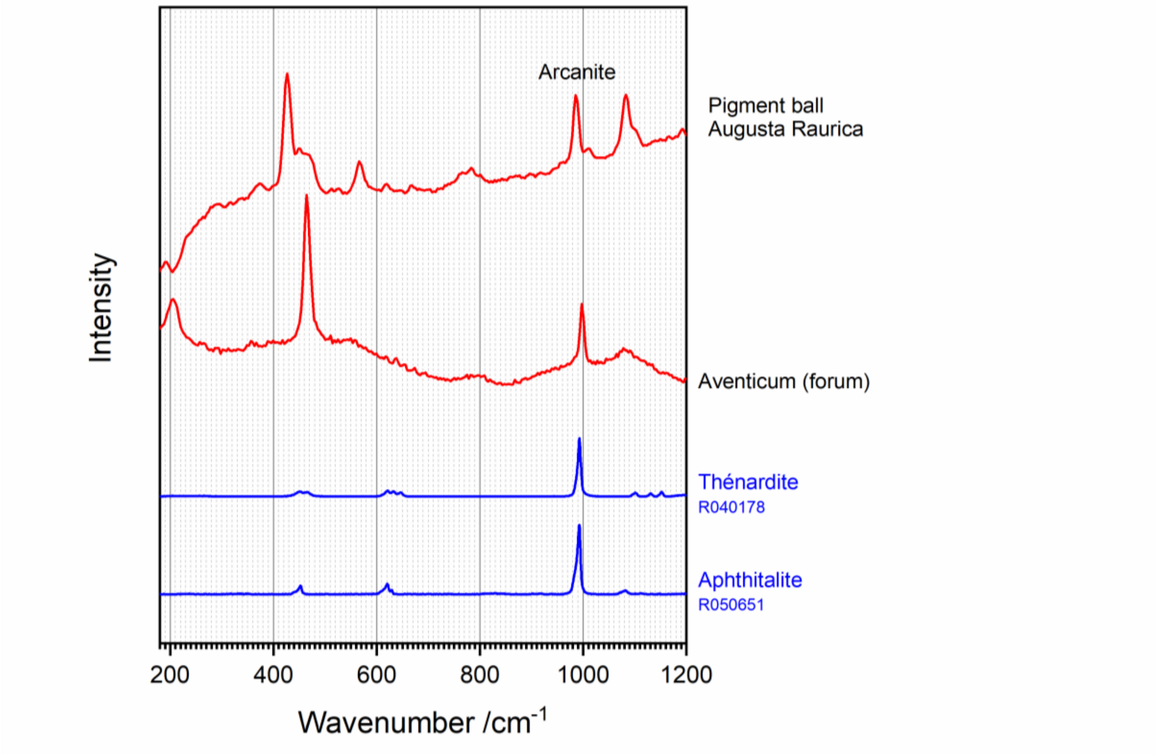

Figure S25: The sulphates arcanite K_2SO_4 [S2], and thénardite Na_2SO_4 or aphthitalite $(K,Na)_3Na(SO_4)_2$.

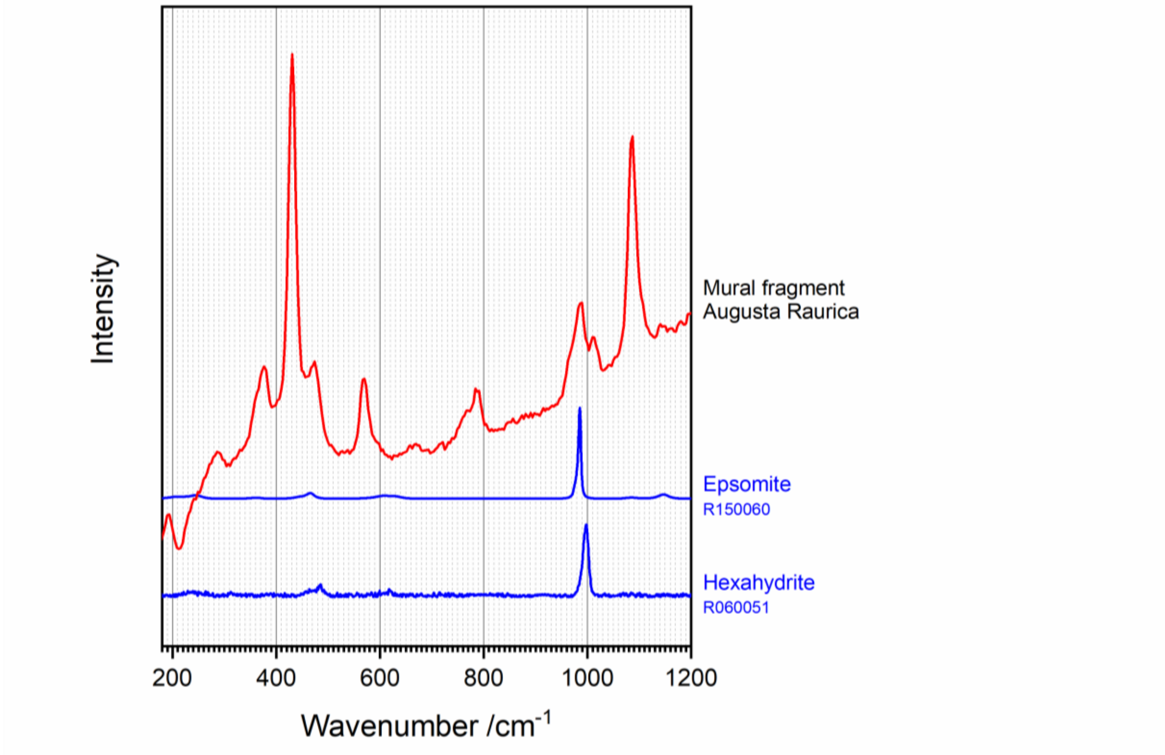

Figure S26: The sulphate epsomite $MgSO_4{\cdot}7H_2O$ and its discrimination from hexahydrite $MgSO_4{\cdot}6H_2O$.

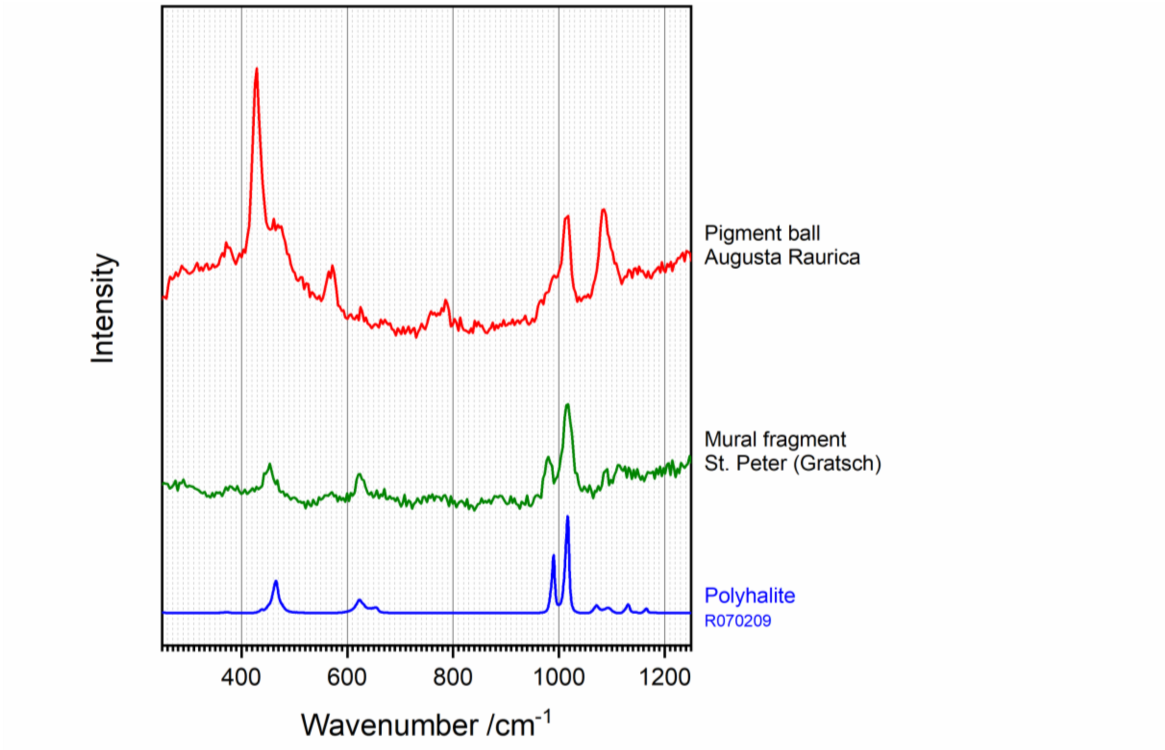

Figure S27: The sulphate polyhalite $K_2Ca_2Mg(SO_4)_4{\cdot}2H_2O$.

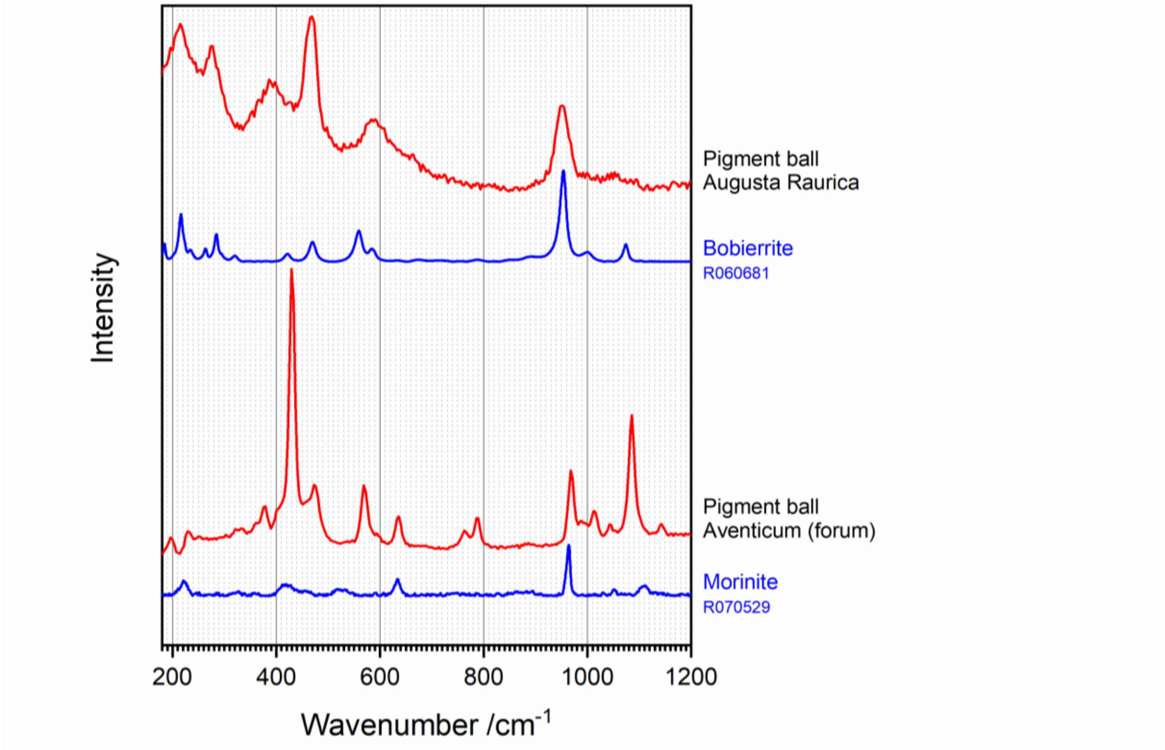

Figure S28: The phosphates morinite $NaCa_2Al_2(PO_4)_2(OH)F_4{\cdot}2H_2O$ and bobierrite $Mg_3(PO_4)_2{\cdot}8H_2O$ (or amorphous calcium phosphate [S3]).

Thermal history of the pigment balls

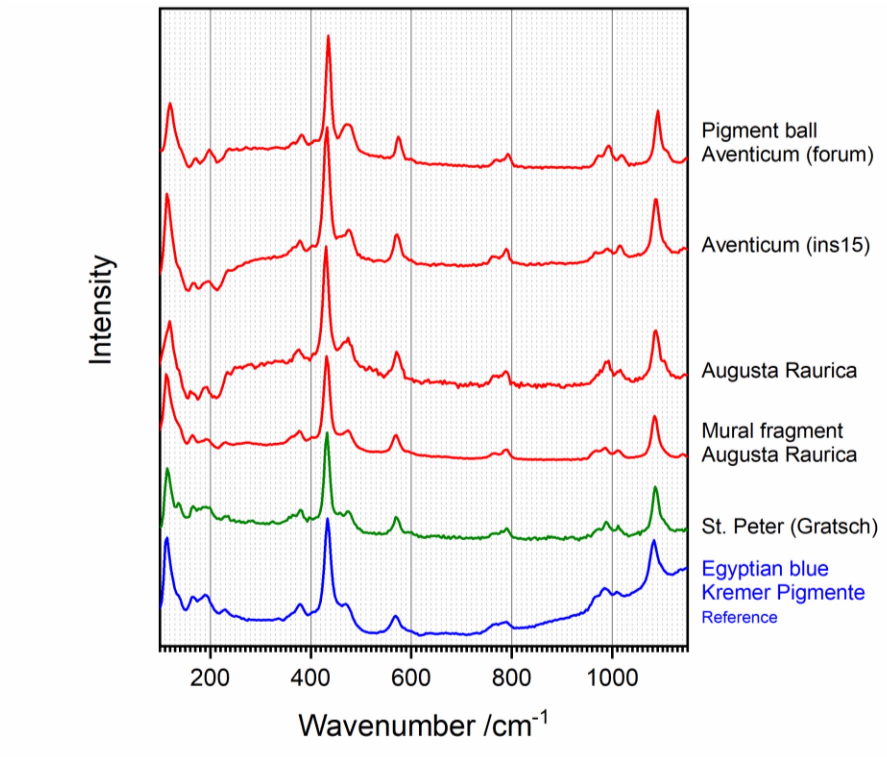

Figure S29: Identification of cuprorivaite in all Roman Imperial (red) and Early Medieval (green) [17] samples under study by comparison of their spectra with reference data from modern Egyptian blue (Kremer Pigmente).

Remarks on the following sections 'Structure and Raman spectrum of cuprorivaite' and 'Disordered cuprorivaite'

During the analysis of the Roman Imperial Egyptian blue samples included in the present study as well as the Early Medieval wall painting fragment discussed in Ref. [17] a comprehensive set of cuprorivaite spectra was acquired (examples shown as blue spectra in the following two sections), which was complemented by reference measurements of a modern Egyptian blue from Kremer Pigmente (Aichstetten, Germany). Data of as-purchased modern cuprorivaite is shown in green, measurement results of the same cuprorivaite finely ground in an agate mortar prior to measurement are diagrammed in red.

Raman band widths and exact band positions in individual spectra – as shown in Figs. S32, S41, and S51 – and deconvolutions of complex spectra into individual peaks (Figs. S39, S40, S44, and S48) were determined by Lorentzian peak fitting [20] by employing the commercial software Origin (OriginLab Corporation, Northampton, MA, USA). For identification of cuprorivaite spectra with baseline-corrected peak intensities high enough for reasonable peak fitting and automated determination of Raman band widths (Lorentz function) and band positions (Gauss function) in full-spectroscopic imaging data consisting of 16 (heating experiments with modern Egyptian blue and muscovite, respectively, shown in Figs. S45 and S52), 10'000 (wall painting Augusta Raurica, Fig. S33) and 1'024 individual spectra (modern Egyptian blue, Fig. S33), respectively, a home-made (T.S.) LabView-based (National Instruments, Austin, TX, USA) software was used. As in this case not every spectrum was manually checked regarding the quality of the result, minor deviations of the fit functions from the acquired spectra may add to the spread of data.

Structure and Raman spectrum of cuprorivaite

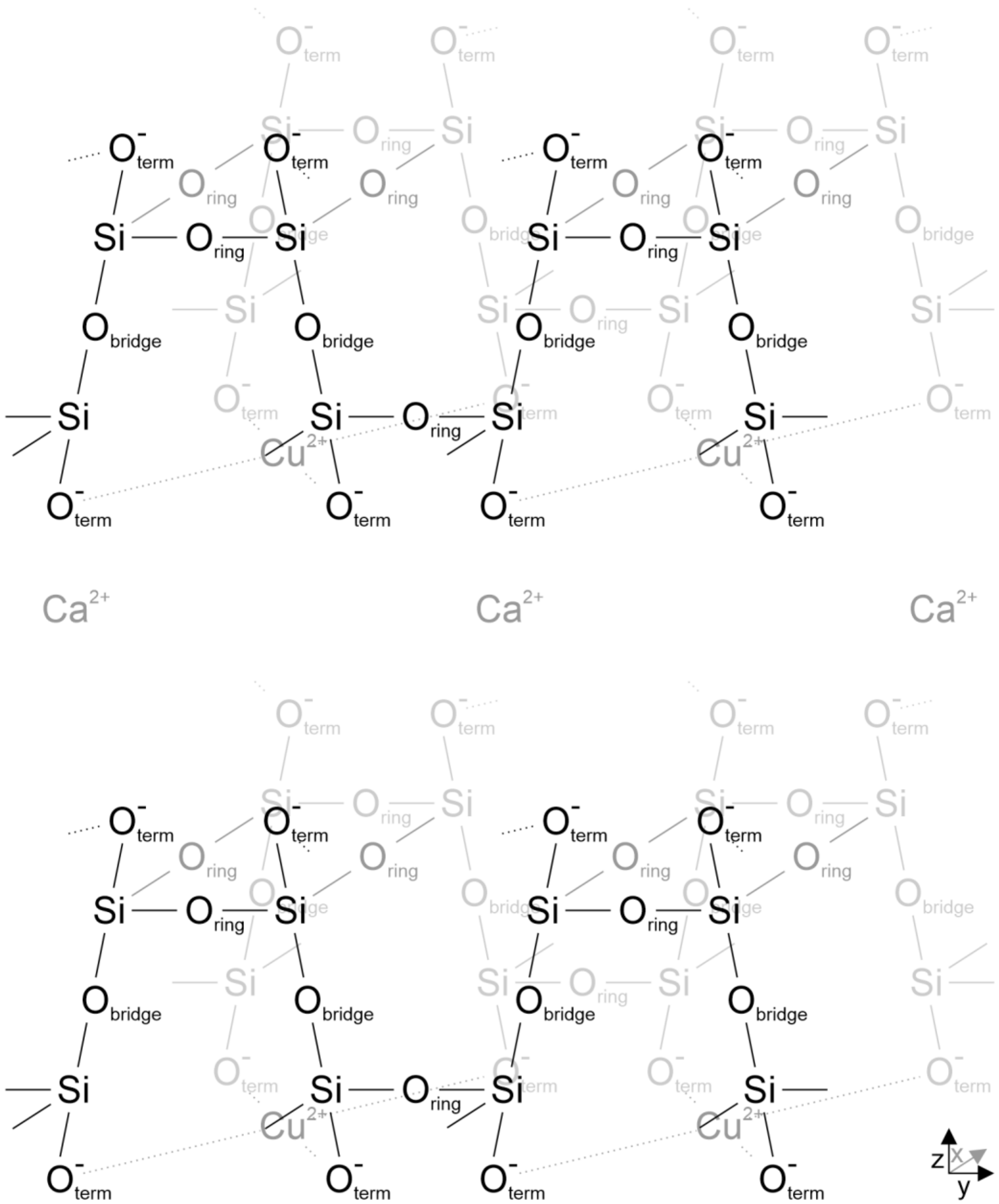

Figure S30: Simplified model of the cuprorivaite structure (see Ref. [53] for realistic representations of the unit cell).

Fig. S30 shows a model of the structure of cuprorivaite, which is simplified in terms of bond lengths and angles with the aim of making structural elements comprehensible, which have a significant effect on the spectroscopic properties of this mineral (see Ref. [53] for realistic representations of cuprorivaite's unit cell). Rings of four silicon atoms interconnected through oxygen atoms (O_{ring} in Fig. S30) are alternatingly arranged in both, x/y and z directions within the structure. Along the z axis, each silicate sheet of this phyllosilicate can be subdivided into a top and a bottom layer, bound together by bridging oxygen atoms (O_{bridge}). The vacancies between silicate rings within the layers are occupied by Cu^{2+} ions that are coordinated by four terminal Si–O^- groups (O_{term}), which also ligate the Ca^{2+} ions interconnecting the silicate sheets. Thus, two Si–O_{ring} bonds of each SiO_4^{4-} tetrahedron span two dimensions of the structure (x/y) and one Si–O_{bridge} adds the third dimension (z), while one Si–O_{term} provides connections to the Cu^{2+} and Ca^{2+} ions. The square planar CuO_4^{6-} complex is the source of both, the blue colour, and the near-

infrared luminescence emission of cuprorivaite [S4]. While according to ligand field theory the isolated CuO_4^{6-} complex appears purple due to optical absorption in the yellow spectral range (see Ref. [S5] for an introduction into ligand field theory in the context of luminescence and Raman spectra), the absorption maximum is shifted to the orange range by the internal electric field created by the whole crystal structure, explaining the typical hue of Egyptian blue [S6].

Theoretically, cuprorivaite and its structural analogues containing different alkaline earth and/or *d*-group ions, such as effenbergerite $BaCuSi_4O_{10}$ (Han blue) and gillespite $BaFeSi_4O_{10}$, have 92 nondegenerate (11 A_{1g} + 12 A_{2g} + 12 B_{1g} + 11 B_{2g} + 12 A_{1u} + 13 A_{2u} + 11 B_{1u} +10 B_{2u}) and 50 doubly degenerate normal modes (25 E_g + 25 E_u), of which one A_{2u} and one E_u are acoustic modes. The A_{1g}, B_{1g}, B_{2g}, and E_g modes are Raman-active, while the A_{2u} and E_u vibrations are infrared-active; the other normal modes are spectroscopically silent [53]. In reality, due to significant changes in relative band intensities depending on the crystal orientation with respect to the laser polarisation direction [11] as well as restricted wavenumber ranges and spectral resolutions of instruments, a single spectrum represents only a subset of the 59 Raman-active modes (11 A_{1g} + 12 B_{1g} + 11 B_{2g} + 25 E_g), which can be roughly subdivided into Si–O stretch vibrations at $\gtrsim$ 800 cm^{-1}, bending/deformation vibrations in an intermediate spectral range, and crystal lattice phonons at $\lesssim$ 140 cm^{-1} [53]. Fig. S31 shows Raman spectra of different crystal orientations of cuprorivaite, and Table S1 provides a comprehensive collection of experimentally determined wavenumbers.

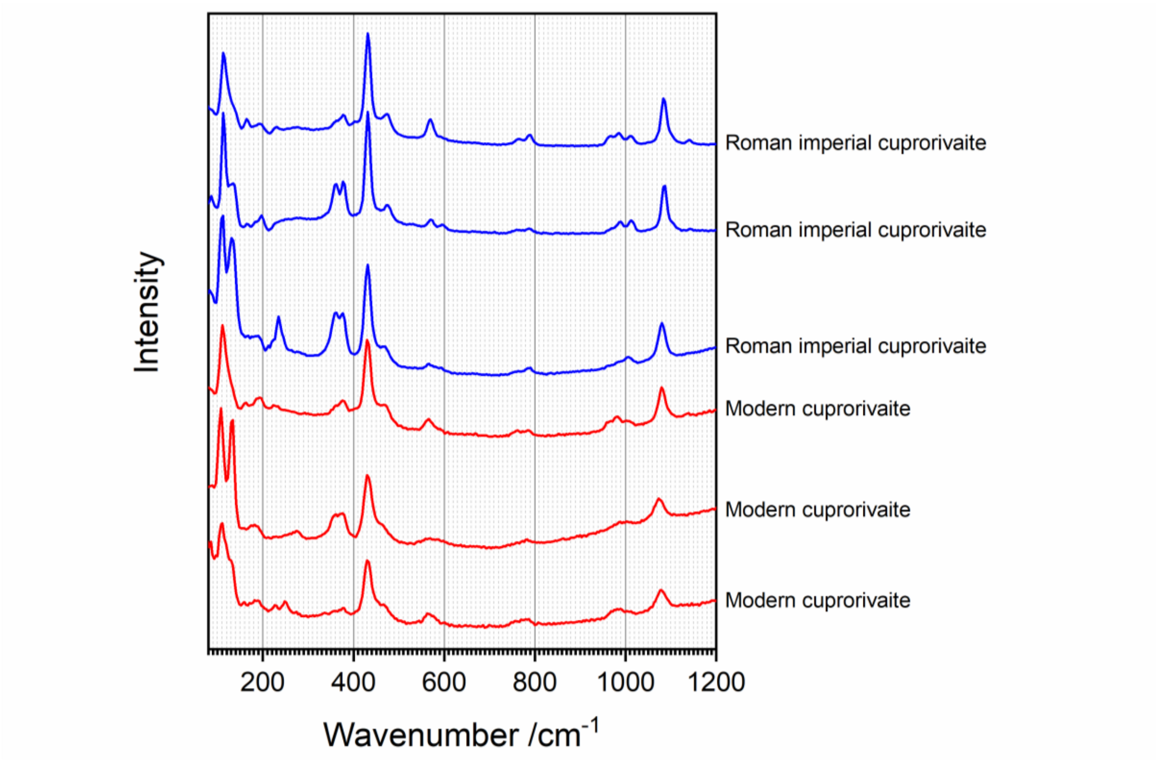

Figure S31: Raman spectra of cuprorivaite selected from datasets of Roman imperial (pictorial layer of the mural fragment from Augusta Raurica) and modern (Kremer Pigmente) Egyptian blue representing different crystal orientations and thus, revealing different relative band intensities.

Table S2. List of experimentally determined Raman wavenumbers of cuprorivaite [11, 14, 66, 17]. Values from the study at hand represent typical crystallinities of Roman Imperial cuprorivaite and may vary due to crystal lattice disorder. This effect is most pronounced for the wavenumbers marked with asterisks (*), as shown in the following section and explained in the main text.

**Cuprorivaite Raman band positions /cm^{-1}**								
J. Raman Spectrosc. **30**, 313-317 (1999).	*Ann. Chim.-Rome* **91**, 679-692 (2001).		*Minerals* **10**, 1063 (2020).		*Sci. Rep.* **11**, 11296 (2021).		Study at hand	
	1141	w	1143	w			1143	w
			1103	w	1102	w (sh)	1102	w (sh)
s	1087	s	1084	s	1083	s	* 1087	s
	1013	w-m	1014	w	1009	m	1013	w-m
	992	w	990	w	986	m	986	w-m
	972	w	967	w	968	m	968	w-m
	788	w-m	788	w	790	m	788	w-m
			776	vw			776	vw
	762	w	762	w	766	m	763	w-m
			613	vw				
	597	vw	595	w	600	w	595	w-m
w-s	572	m	570	w	569	m	* 570	w-m
							545	vw
	522	vw						
w-m	477	m	475	w	469	m (sh)	* 473	m (sh)
vs	432	vs	430	vs	433	vs	432	vs
w-vs	378	m	378	s	379	m	378	w-vs
w-vs	361	w-m	359	s	361	w	361	w-vs
	340	vw			336	vw	336	vw
							274	vw
			247	vw			248	w
w-m	231	w-m	231	vw	229	w	234	w-m
							226	vw-w
			215	vw				
w	198	m	196	w	192	m	193	w-m
			186	vw			183	vw
w-m	165	w	163	vw	166	m	163	w-m
m-vs	137	s	138	s	137	m	131	m-vs
m-vs	114	ms	113	s	114	s	* 114	m-vs

Table S3. Assignments of the Raman-active vibrational modes of cuprorivaite discussed in this article.

**Raman band of cuprorivaite $CaCuSi_4O_{10}$**	**Assignment**	**Analogue band(s) of gillespite $BaFeSi_4O_{10}$ [53]**
1087 cm^{-1}	Si–O_{bridge}–Si stretching vibration [14]	1092 cm^{-1} B_{1g} Si–O_{bridge}–Si stretch
788 cm^{-1}	ν_c mode [14], ring deformation vibration [74]	789 cm^{-1} B_{1g} ν_c mode
570 cm^{-1}	O_{term}–Si–O_{bridge} bending mode [14]	558 cm^{-1} B_{1g} O_{term}–Si–O_{bridge} bending
473 cm^{-1}	Si–O_{term}/Si–O_{bridge} rocking mode [14]	522 cm^{-1} E_g Si–O_{term}/Si–O_{bridge} rocking
432 cm^{-1}	combination of deformation [14] and breathing modes of silicate rings	427 cm^{-1} B_{1g} ν_c+O_{ring} z translation;<br>427 cm^{-1} E_g O_{ring} z translation and Si xy translation;<br>450 cm^{-1} A_{1g} O_{ring} breathing
114 cm^{-1}	motion of Ca^{2+} relative to the silicate structure [14]	102 cm^{-1} E_g Ba^{2+} x- and y-translation

Disordered cuprorivaite

(a)

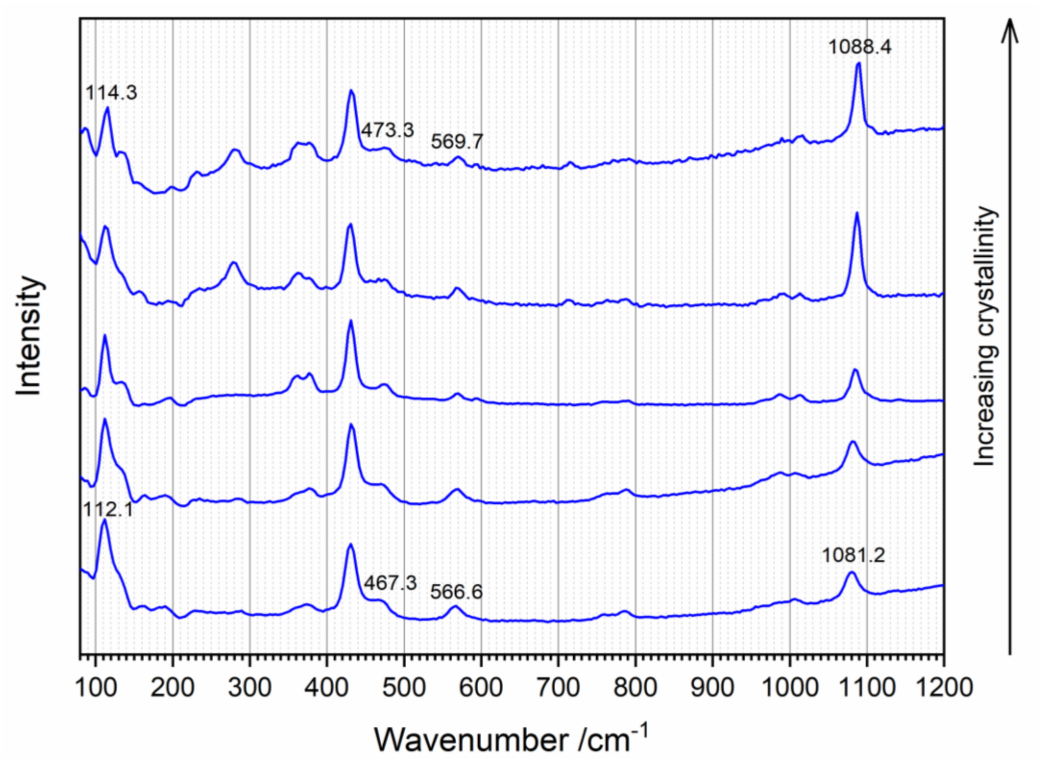

(b)

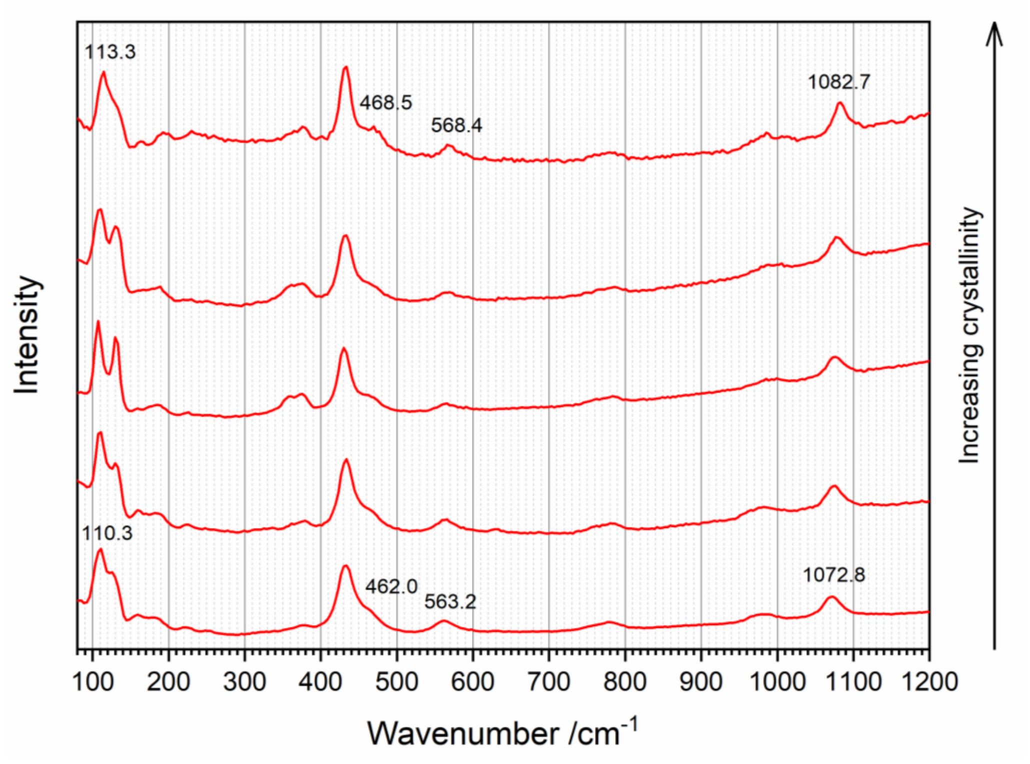

Figure S32: Different crystallinities of Roman Imperial (pictorial layer of the mural fragment from Augusta Raurica) **(a)** and modern cuprorivaite (Kremer Pigmente) **(b)**.

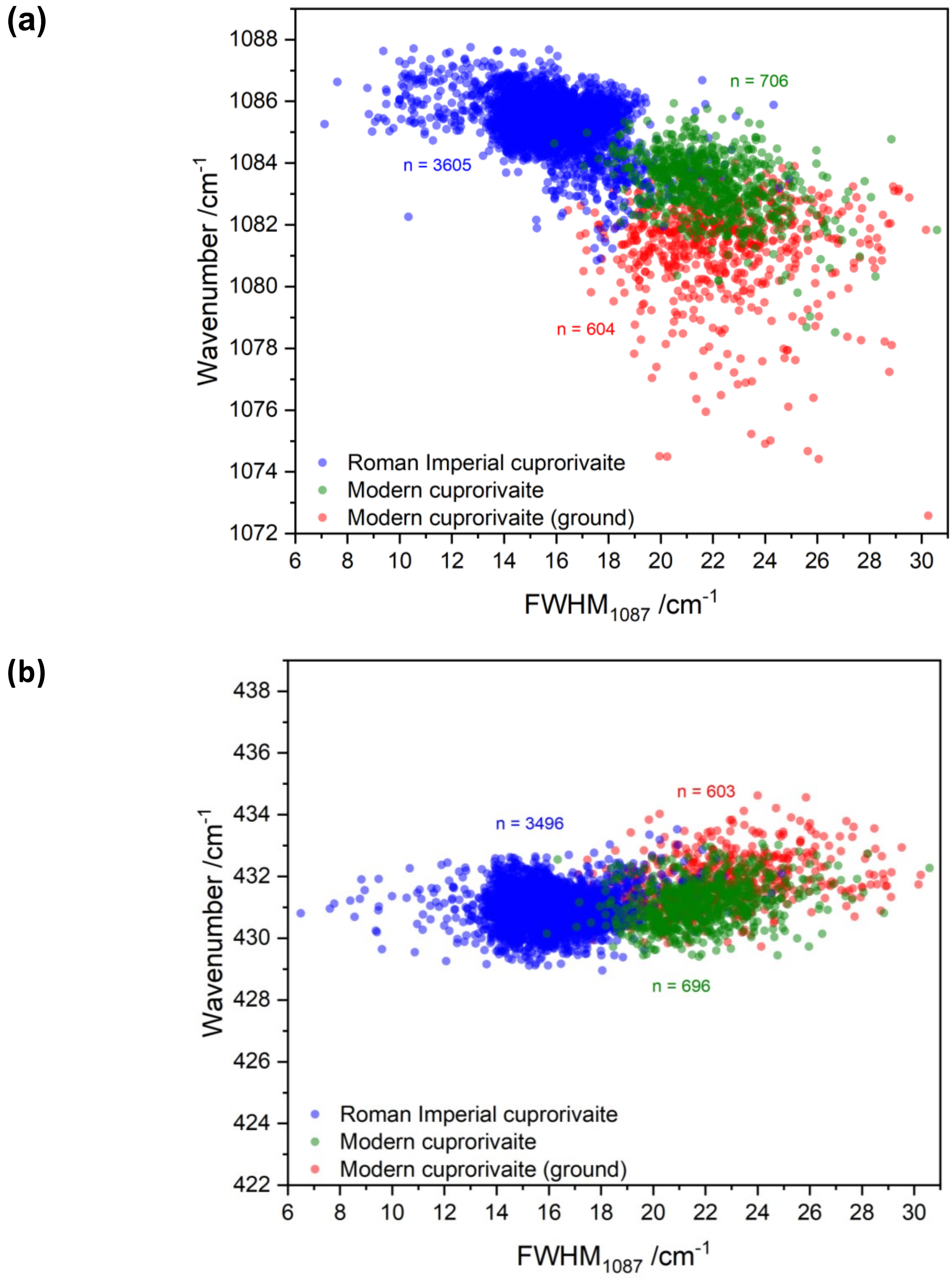

Figure S33: Correlations between the band position of the 1087-cm^{-1} **(a)**, the 432-cm^{-1} mode **(b)** of cuprorivaite and the width (expressed as full width at half maximum or FWHM, respectively) of the 1087-cm^{-1} band. Samples: pictorial layer of the mural fragment from Augusta Raurica (Roman Imperial cuprorivaite), and modern Egyptian blue from Kremer Pigmente (pristine or finely ground in an agate mortar); n denotes the number of evaluated spectra.

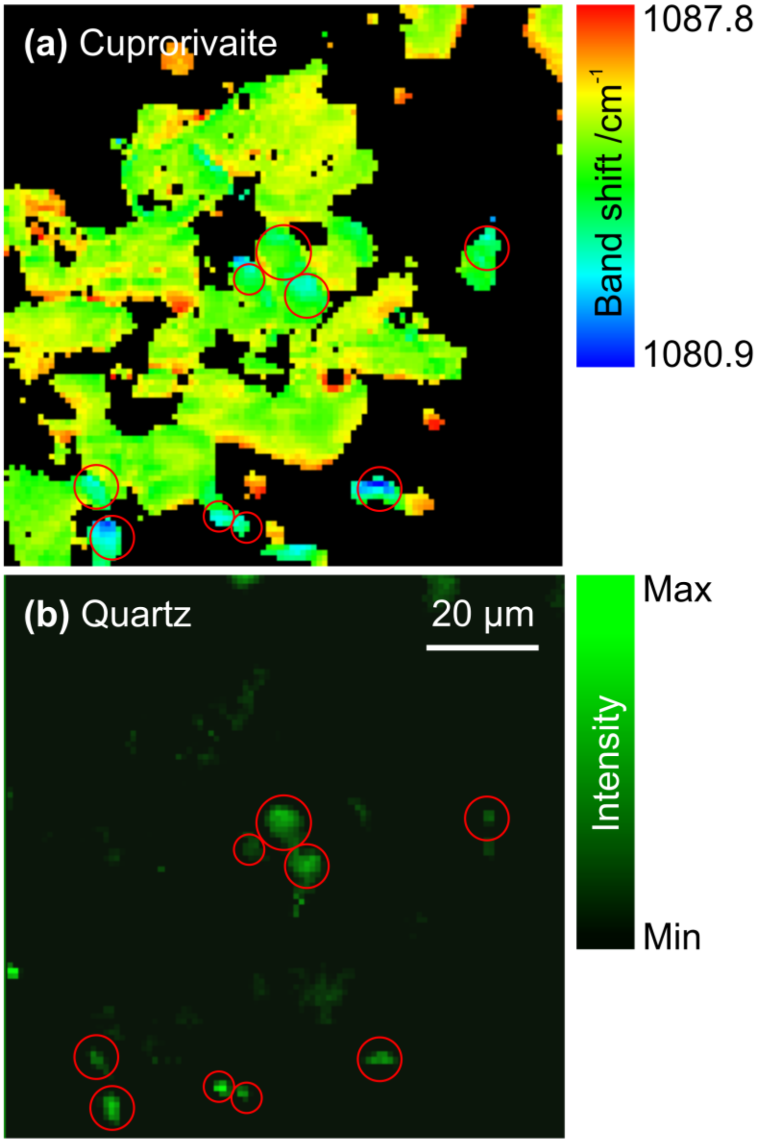

Figure S34: Distribution and shift of the 1087-cm^{-1} band of cuprorivaite **(a)**, and distribution of quartz based on the baseline-corrected peak height of the Raman band at 465 cm^{-1} **(b)**. The red circles were added as guide to the eye, revealing co-localised disordered cuprorivaite (characterised by band shifts to lower wavenumbers, indicated by blue to cyan false colours) and quartz. This figure represents alternative evaluations of the data set of the pictorial layer of the mural fragment from Augusta Raurica shown in Fig. 3 (right) in the main text.

By-products of the cuprorivaite synthesis: Wollastonite and green glass

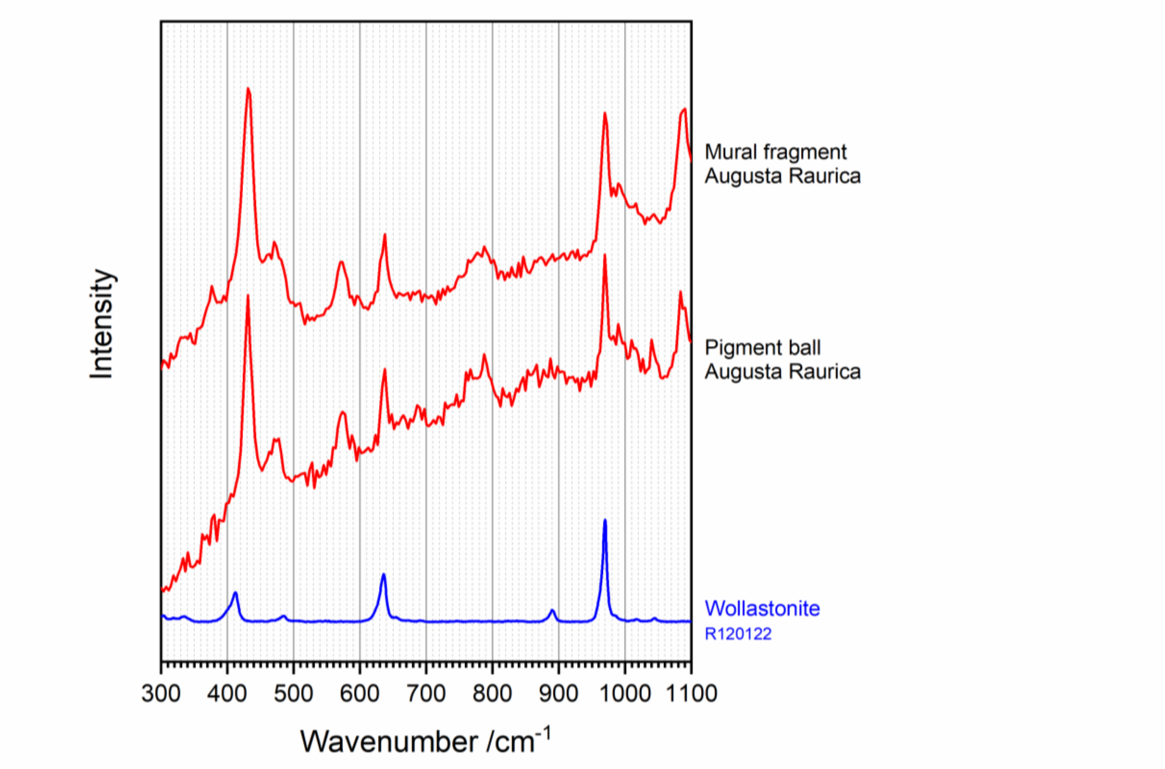

Figure S35: The pyroxenoid wollastonite $CaSiO_3$.

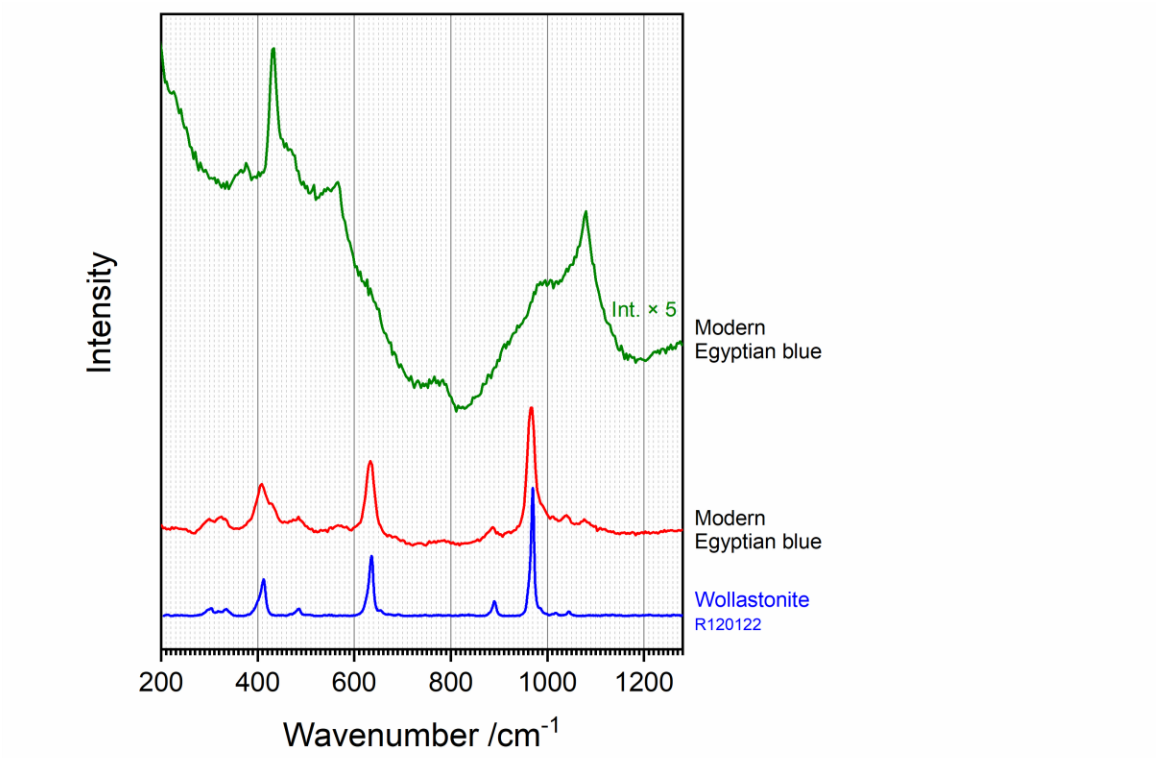

Figure S36: By-products of the synthesis in modern cuprorivaite (Kremer Pigmente): green glass phase (green trace), and wollastonite (red trace) compared to reference data from the RRUFF spectral library (blue trace) [19].

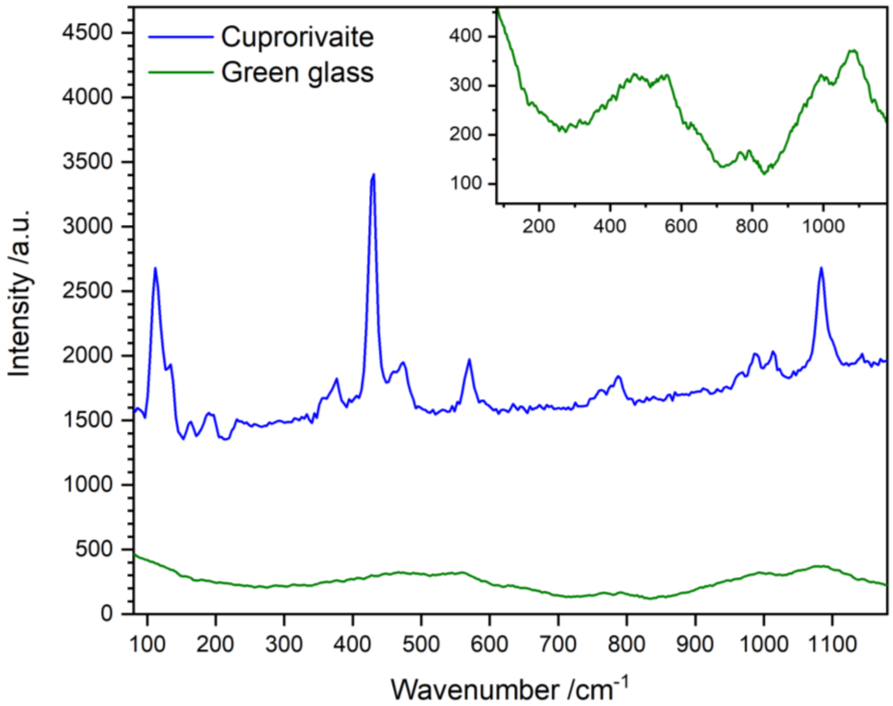

Figure S37: Spectra of cuprorivaite and green glass phase from the same Raman map of the pigment sphere Aventicum (forum) demonstrating their different Raman scattering intensities.

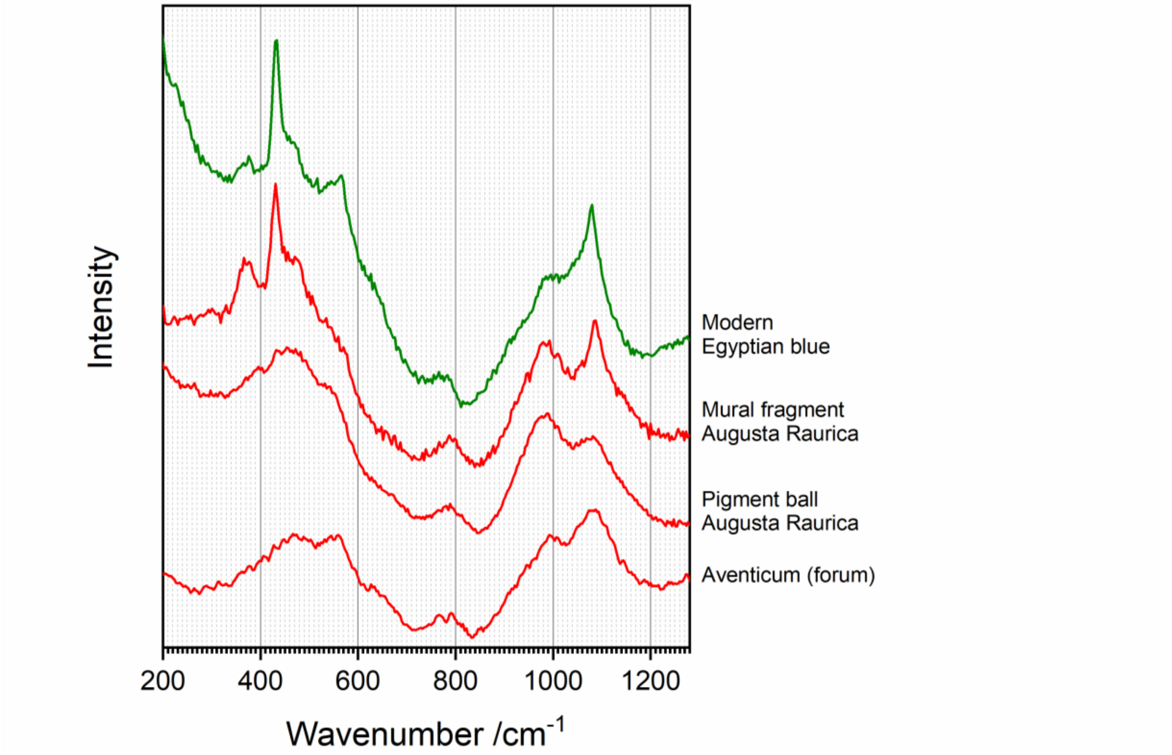

Figure S38: Raman spectra of a green glass phase locally found in modern (Kremer Pigmente) and Roman Imperial Egyptian blue (pictorial layer of the mural fragment from Augusta Raurica, as well as pigment balls from Aventicum (forum) and Augusta Raurica). Sharp bands in the two uppermost spectra are due to co-localised cuprorivaite. Deconvolutions of the pure glass spectra acquired on the pigment balls are shown in Figs. S39 and S40.

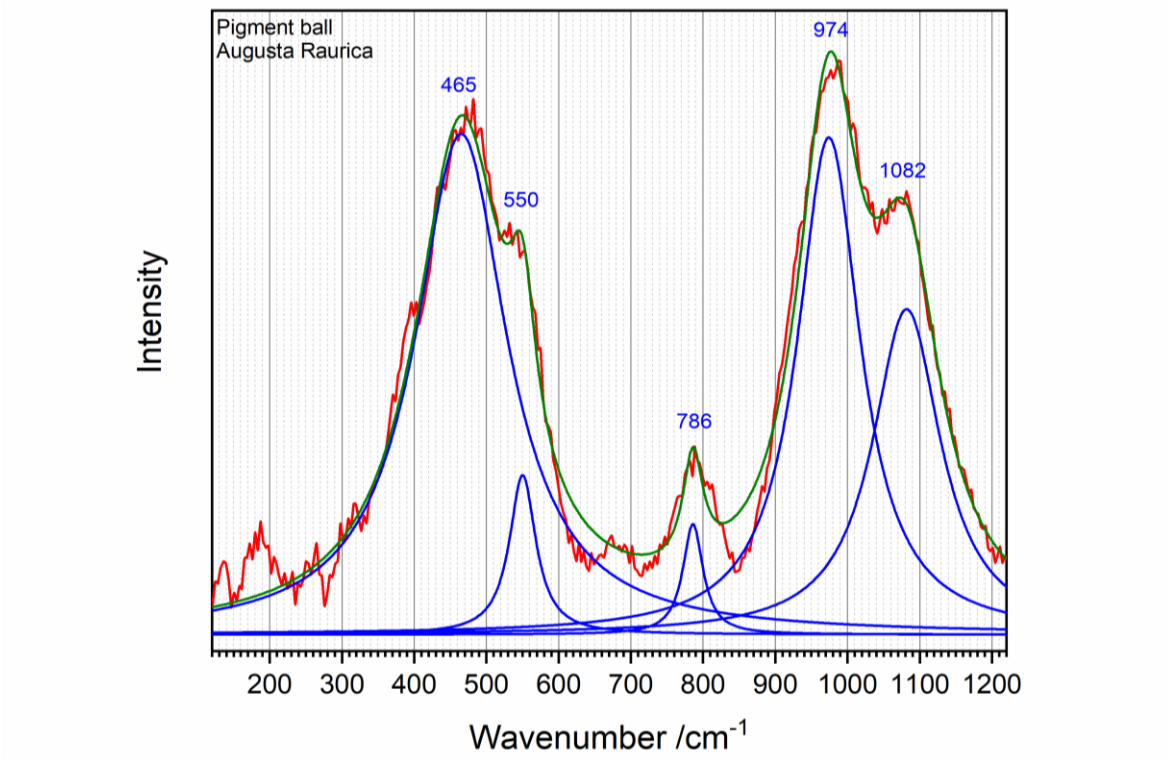

Figure S39: Raman spectrum of a green glass phase found in the pigment ball from Augusta Raurica, intensity-corrected (intensity correction system, ICS), baseline-corrected, and deconvoluted into Lorentzian peaks.

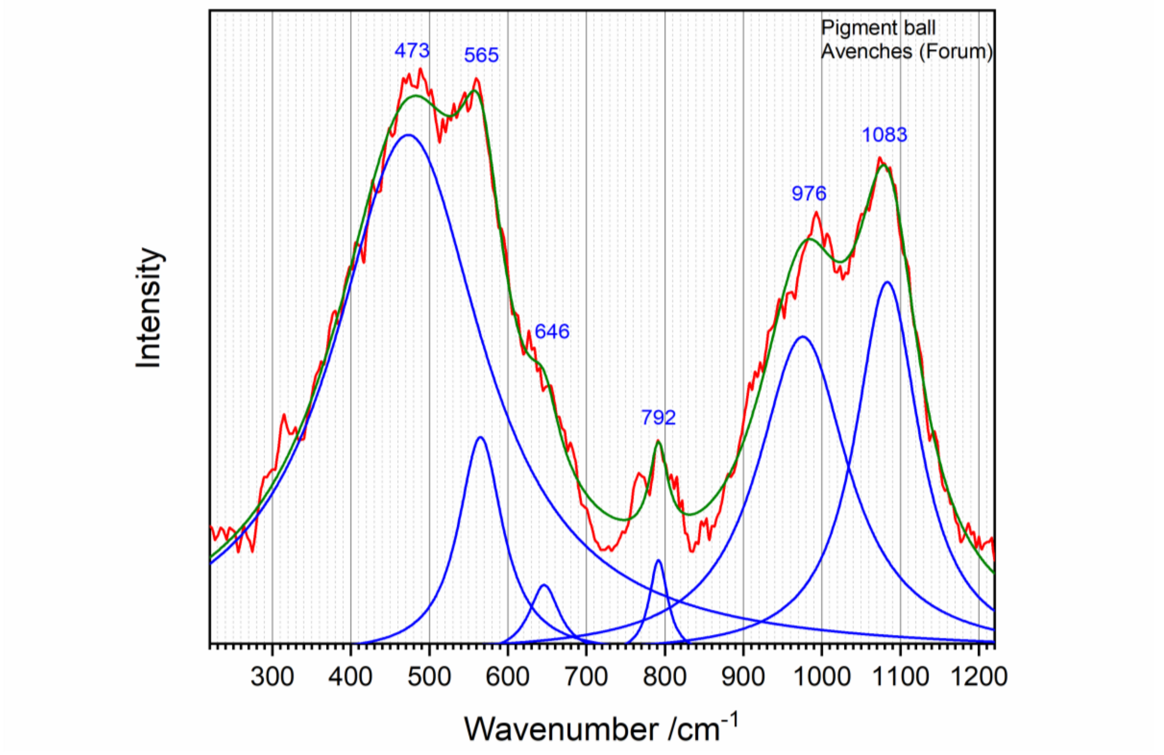

Figure S40: Raman spectrum of a green glass phase found in the pigment ball from Aventicum (forum), intensity-corrected (intensity correction system, ICS), baseline-corrected, and deconvoluted into Lorentzian peaks.

The Raman spectrum of the green glass phase observed in both Roman Imperial and modern Egyptian blue shows variations in band positions and relative intensities in the low-wavenumber range, a mid-range massif at around 785 cm^{-1} and fairly reproducible peaks at approx. 975 cm^{-1} and 1085 cm^{-1} in the high-wavenumber region (see Figs. S39–S40), the latter three resembling very well positions of bands or multiplets of cuprorivaite, opening up a possible interpretative approach (see illustration of the similarities and differences of the Raman spectra of this amorphous phase, disordered and crystalline cuprorivaite in Fig. 4 in the main text): David A. McKeown and Michael I. Bell [53] compared the wavenumbers of features characterising Raman spectra of SiO_2 glass with band positions of gillespite and confirmed their theoretically calculated assignment to vibrational modes of four-membered silicate rings in the glass structure [S7]. As a further analogy, we interpret the massif between approx. 750 cm^{-1} and 800 cm^{-1} in the spectra of the green glass phase found in Egyptian blue as vibrations of rings consisting of four interconnected SiO_4^{4-} tetrahedra, which coordinate Cu^{2+} ions. For cuprorivaite (based on the interpretation of the gillespite spectrum [53]), the band at 788 cm^{-1} was assigned to the ν_c vibrational mode [14], which like bands in the same wavenumber range is explained as a ring deformation motion [74]. In analogy to the gillespite spectrum, exhibiting these bands at only slightly shifted positions, such massif in a glass spectrum might be induced by other heteroelement ions such as Fe^{2+} as well, but as the spectroscopically and chromatically concordant glass phase was also detected as a by-product in modern cuprorivaite synthesised from pure starting materials (see Figs. S36 and S38), its correlation in the spectra at hand with Cu^{2+} becomes obvious.

Thermal decomposition experiment of modern Egyptian blue

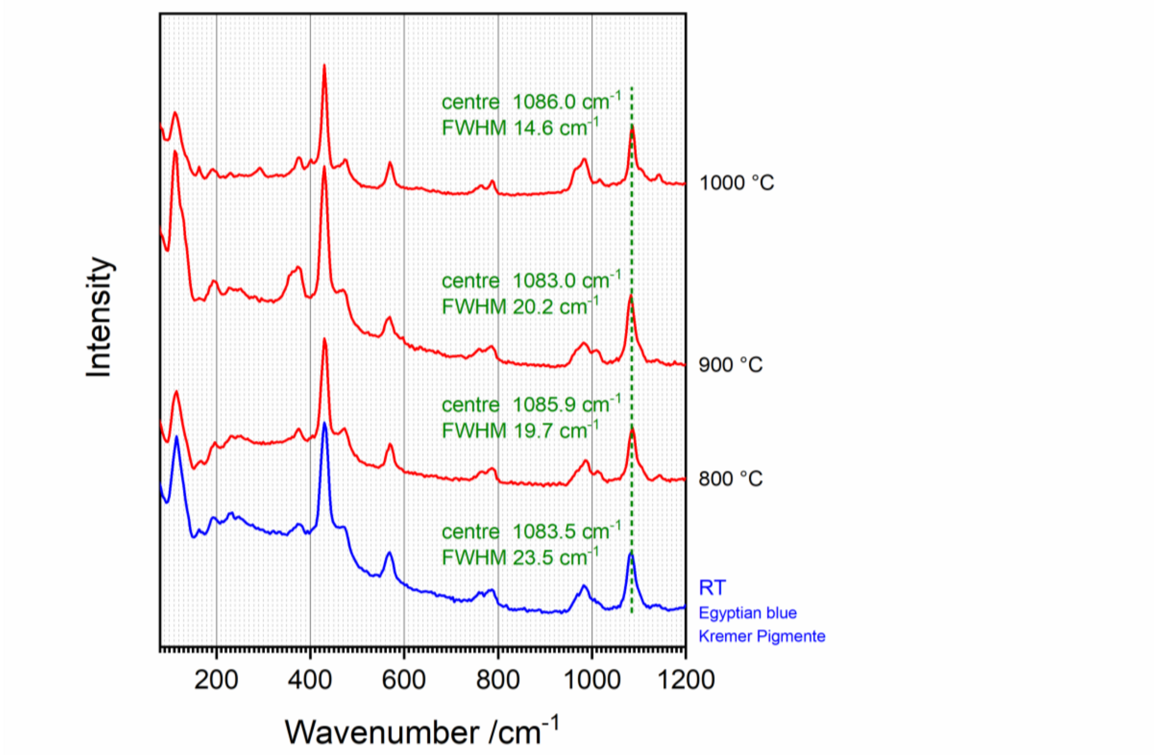

Figure S41: Thermal decomposition of modern Egyptian blue (Kremer Pigmente): 800–1000 °C.

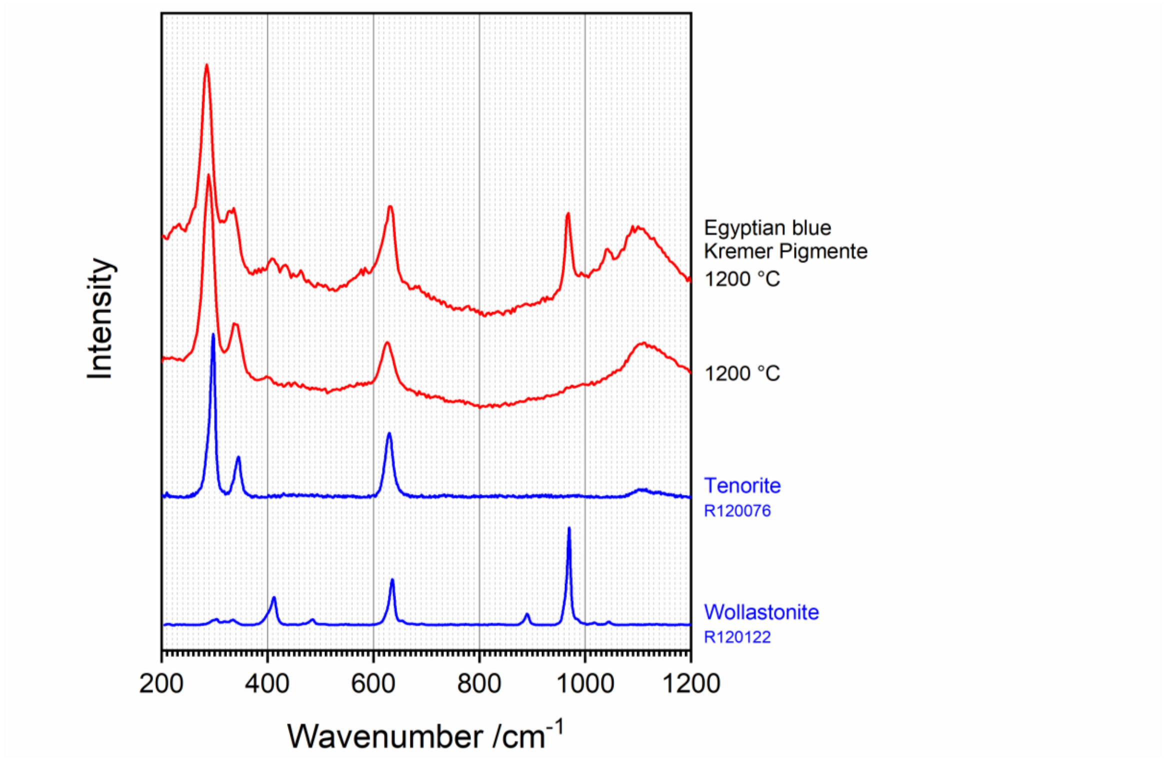

Figure S42: Thermal decomposition of modern Egyptian blue (Kremer Pigmente): 1200 °C. Wavenumber downshift compared to the reference spectrum and band broadening of tenorite may be due to the nanometric size of the thermally generated CuO [S8–S9].

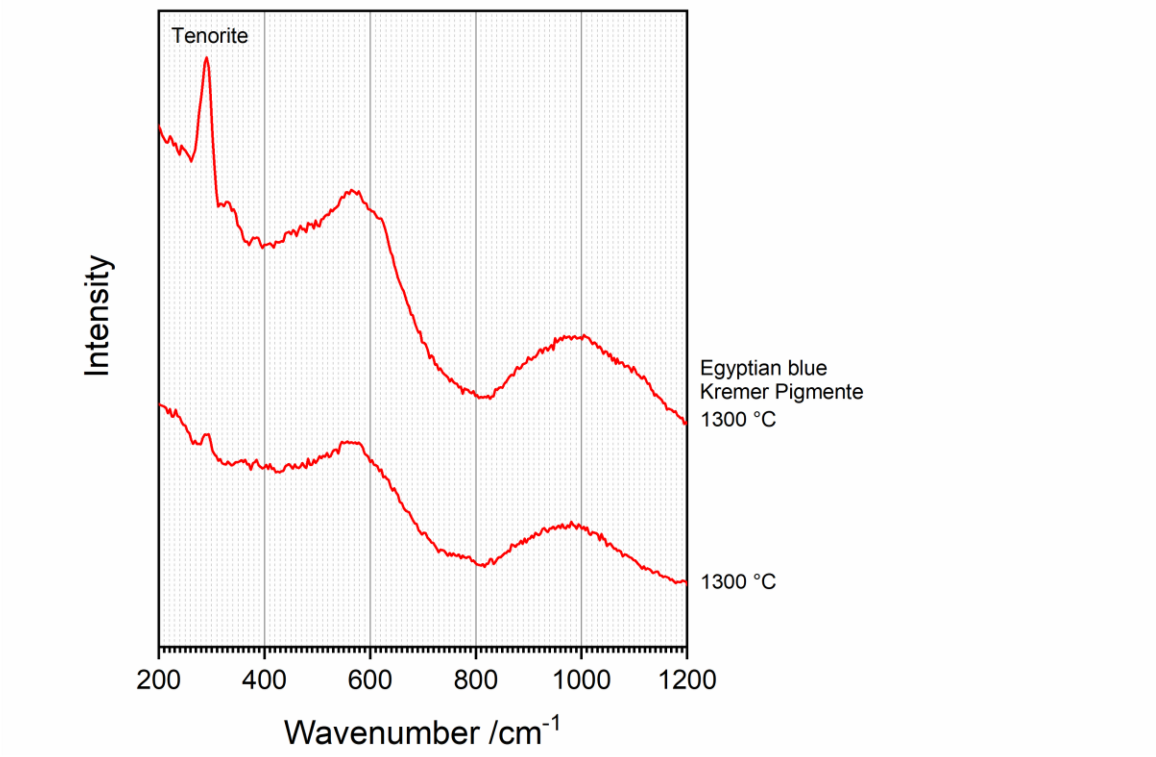

Figure S43: Thermal decomposition of modern Egyptian blue (Kremer Pigmente): 1300 °C.

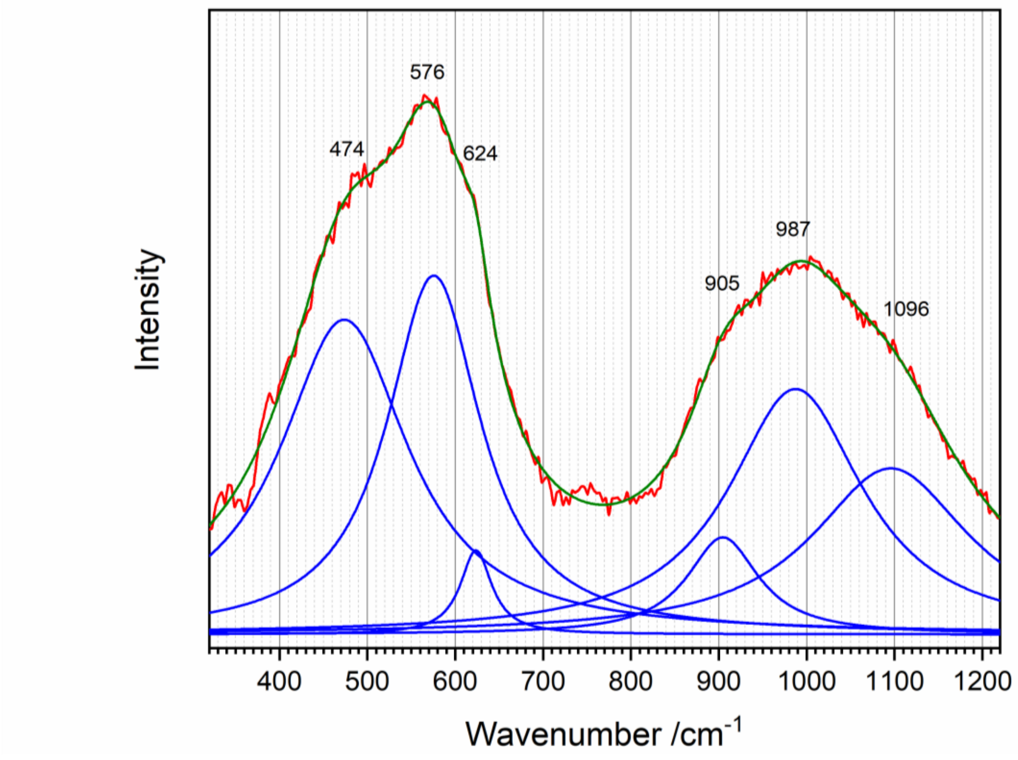

Figure S44: Raman spectrum of a colourless copper-free glass phase (mixed with dark-coloured tenorite) produced from modern Egyptian blue (Kremer Pigmente) at 1300 °C, intensity-corrected (intensity correction system, ICS), baseline-corrected, and deconvoluted into Lorentzian peaks. Note the different wavenumbers and the missing mid-range bands in comparison to the spectrum of the copper-bearing green glass phase found as a by-product in Egyptian blue (Figs. S39–S40).

Thermal decomposition experiment of modern Egyptian blue with $NaHCO_3$ flux

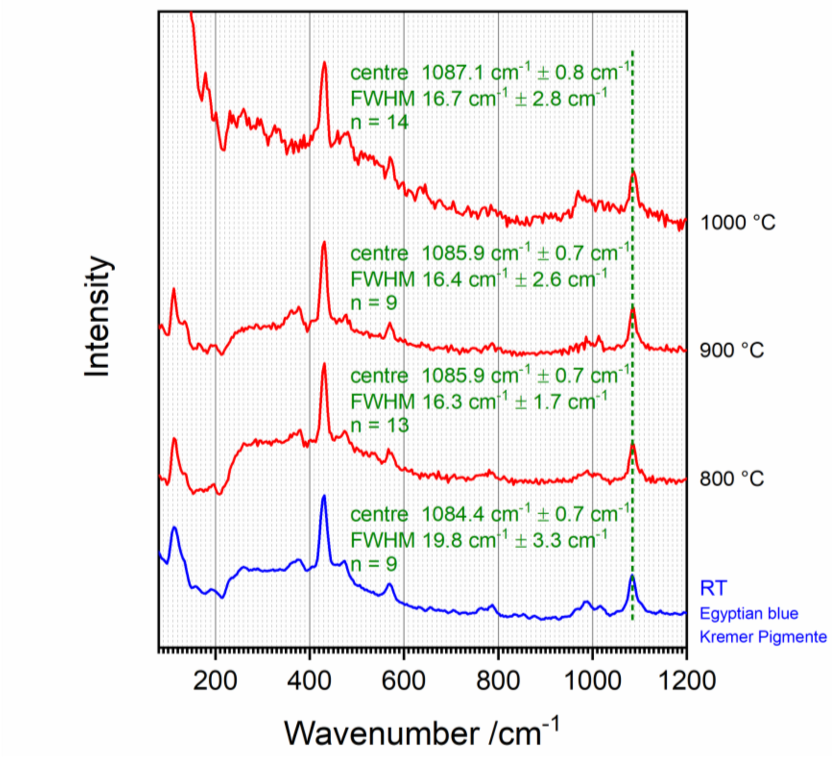

Figure S45: Thermal decomposition of modern Egyptian blue (Kremer Pigmente) mixed with $NaHCO_3$ flux in a molar ratio of 3:1: 800–1000 °C.

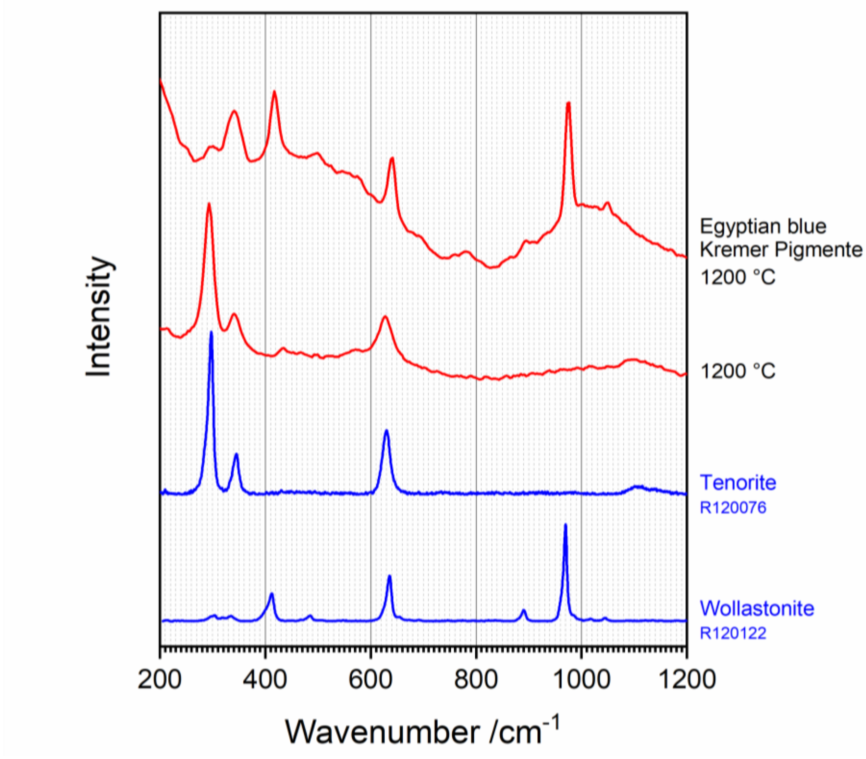

Figure S46: Thermal decomposition of modern Egyptian blue (Kremer Pigmente) mixed with $NaHCO_3$ flux in a molar ratio of 3:1: 1200 °C. Wavenumber downshift compared to the reference spectrum and band broadening of tenorite may be due to the nanometric size of the thermally generated CuO [S8–S9].

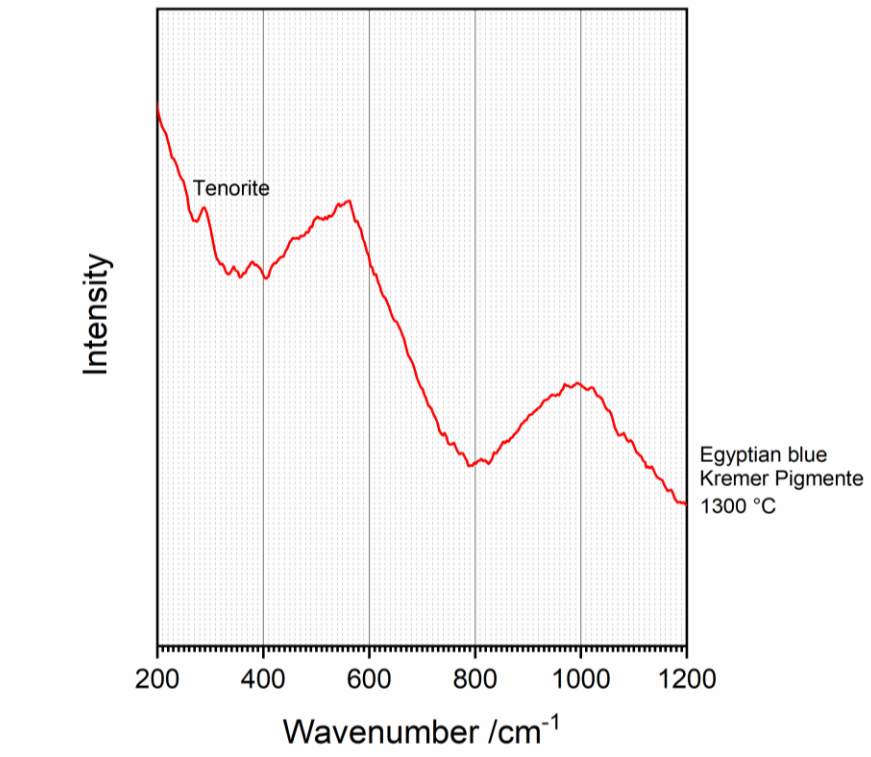

Figure S47: Thermal decomposition of modern Egyptian blue (Kremer Pigmente) mixed with $NaHCO_3$ flux in a molar ratio of 3:1: 1300 °C.

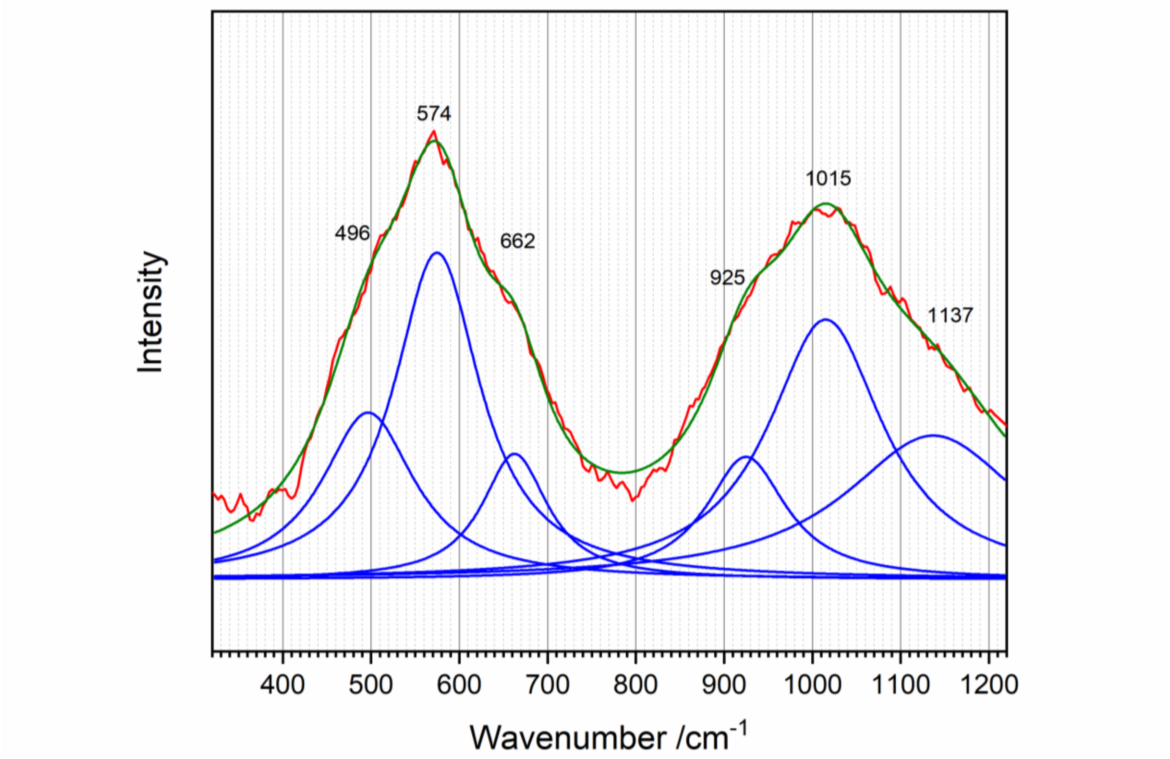

Figure S48: Raman spectrum of a colourless copper-free glass phase (mixed with dark-coloured tenorite) produced from modern Egyptian blue (Kremer Pigmente), mixed with $NaHCO_3$ flux in a molar ratio of 3:1, at 1300 °C, intensity-corrected (intensity correction system, ICS), baseline-corrected, and deconvoluted into Lorentzian peaks. Note the different wavenumbers and the missing mid-range bands in comparison to the spectrum of the copper-bearing green glass phase found as a by-product in Egyptian blue (Figs. S39–S40).

Contaminations by adherent soil minerals

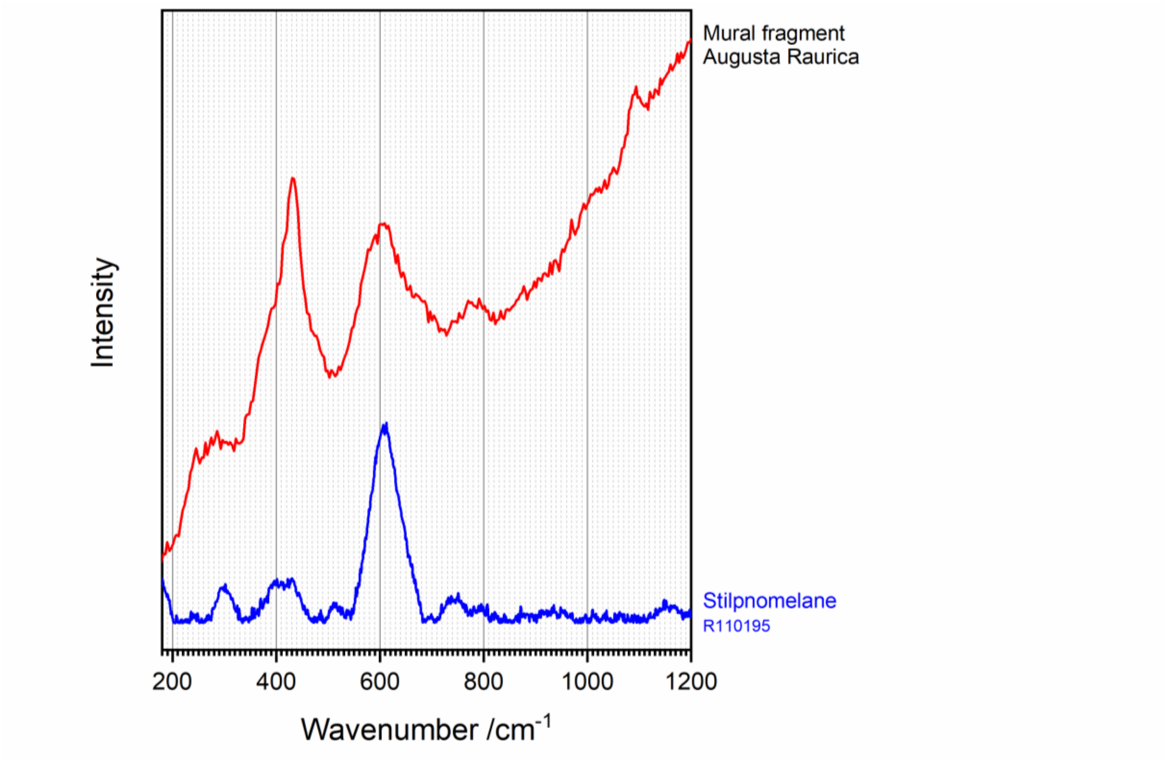

Figure S49: The phyllosilicate stilpnomelane $K(Fe,Mg,Al)_8(Si,Al)_{12}(O,OH)_{27}\cdot 2H_2O$.

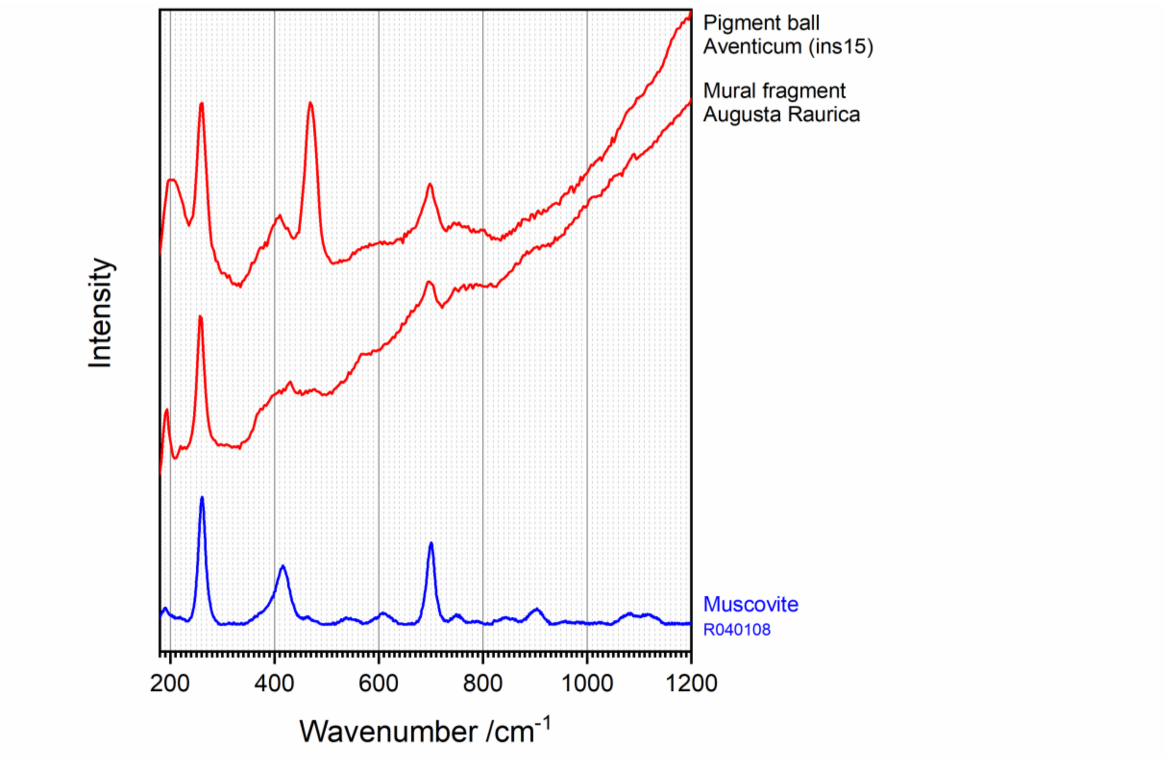

Figure S50: The phyllosilicate muscovite $KAl_2(Si_3Al)O_{10}(OH,F)_2$.

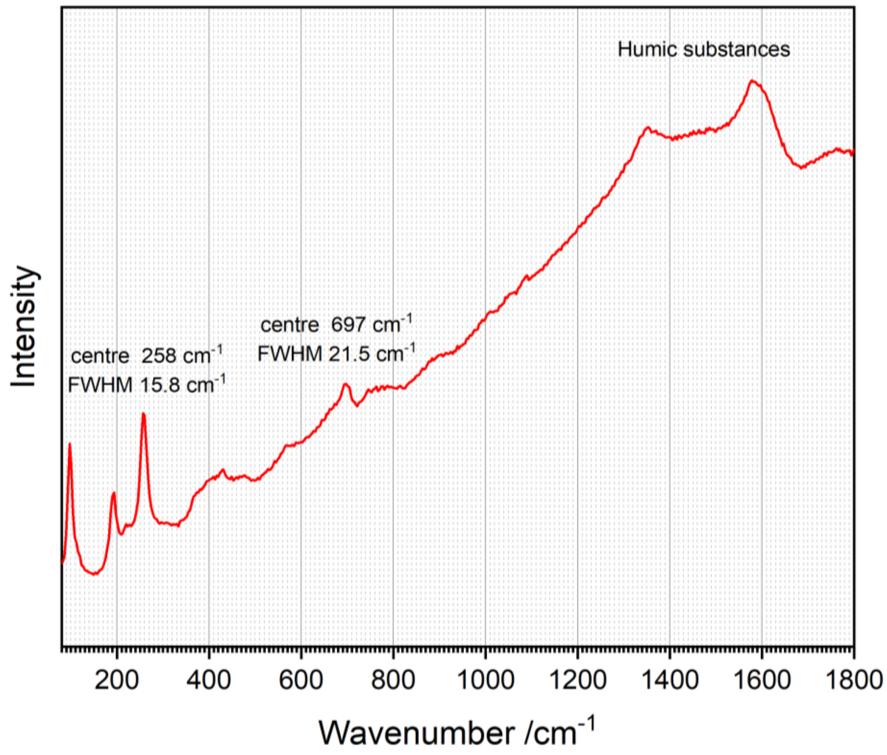

Figure S51: Raman spectrum of muscovite (pictorial layer of the mural fragment from Augusta Raurica) shown in a wider spectral range than in Fig. S50, also including general indicators of sample contamination by soil constituents: Raman spectrum of humic substances [78] and according autofluorescence emission causing a sloped baseline. The peak centres and widths yielded by Lorentzian fitting speak against thermal damage (compare Fig S52 below).

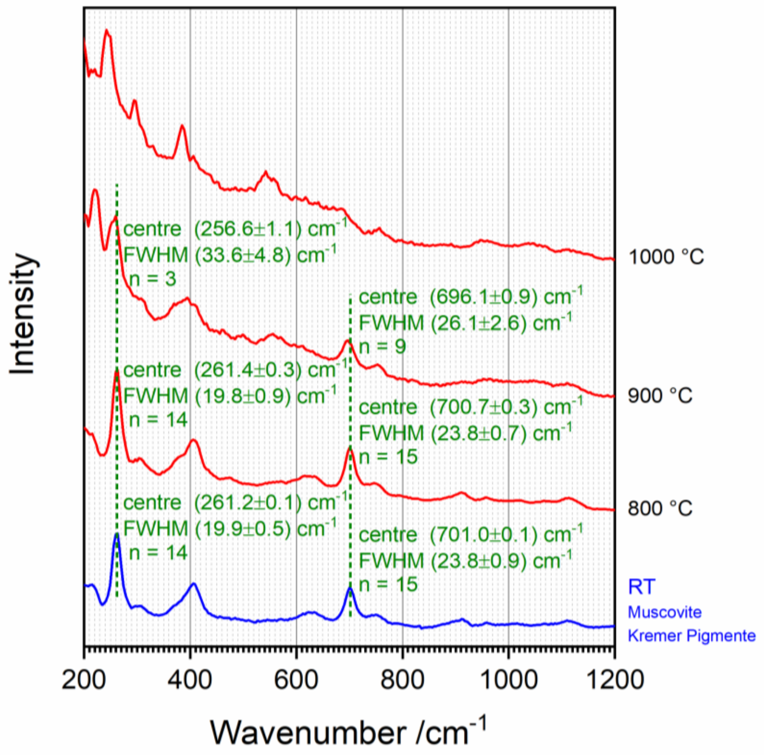

Figure S52: Thermal decomposition experiment of muscovite (Kremer Pigmente, Aichstetten, Germany).

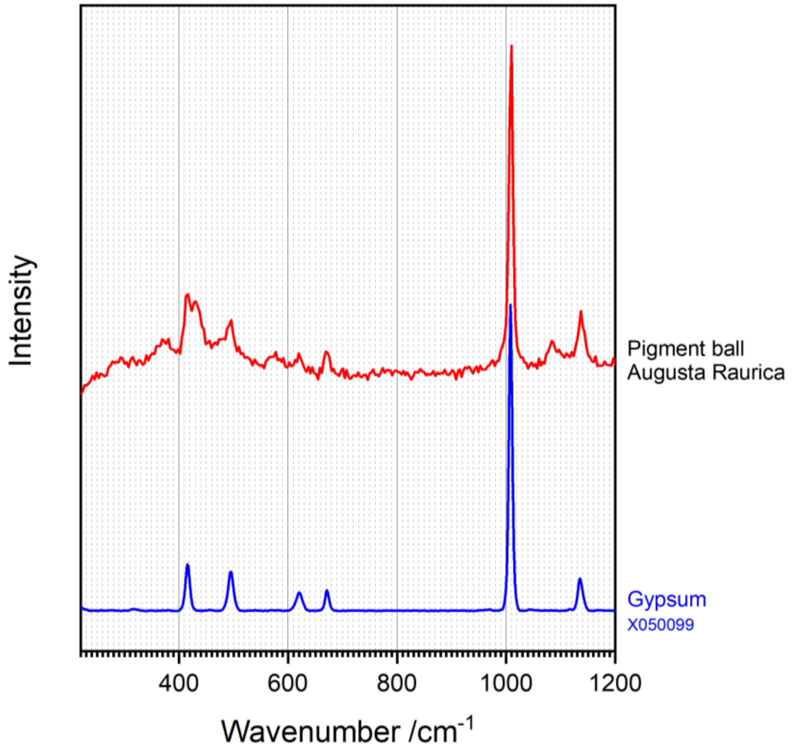

Figure S53: Gypsum $CaSO_4 \cdot 2H_2O$.

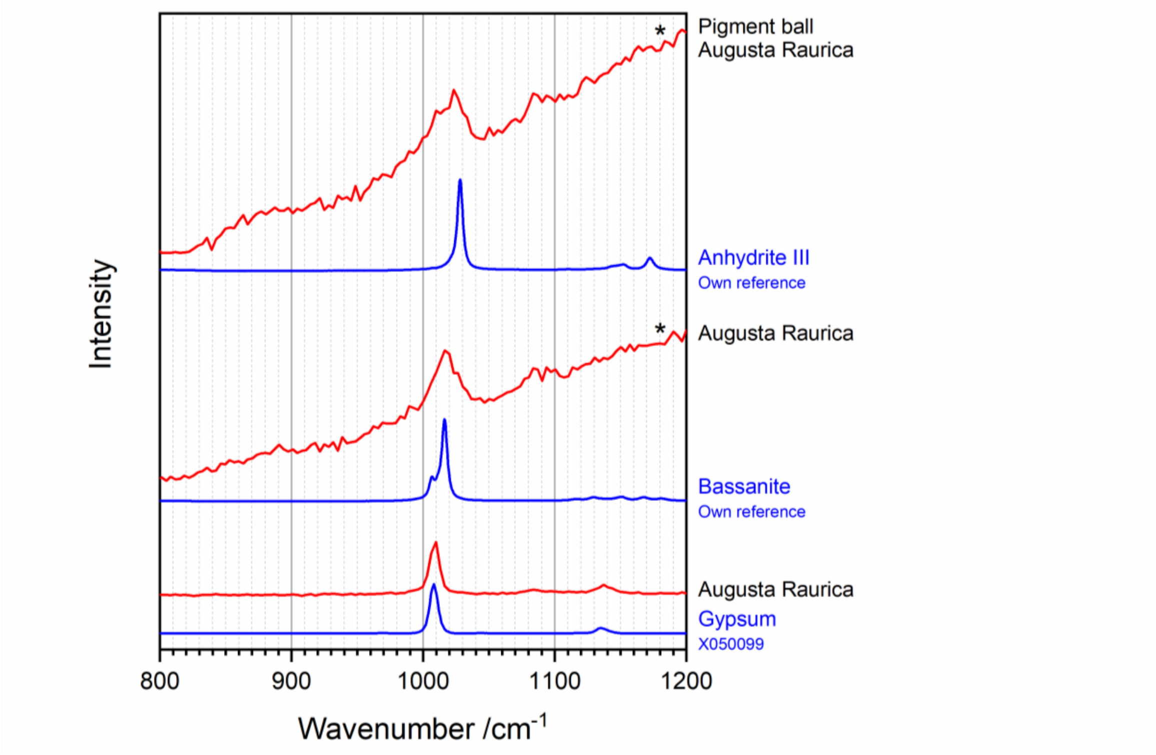

Figure S54: Gypsum on the surface of pigment ball Augusta Raurica. Spectra marked with an asterisk (*) show the results of local heating due to absorption of the laser beam employed for the Raman measurements by organic chromophores in the form of humic substances and/or other soil constituents. Reference data are either from the RRUFF database library [17] or from Ref. [85].

References

Citations in the text above of the form [n] (with n denoting a number) refer to the list of references in the main text, while literature exclusively cited within the Supplementary Information is designated as [Sn] and listed as follows.

S1. Pelosi, C., Agresti, G., Santamaria, U. & Mattei, E., Artificial yellow pigments: Production and characterization through spectroscopic methods of analysis. *e-PS* **7**, 108–115 (2010).

S2. Buzgar, N., Buzatu, A. & Sanislav, I. V., The Raman study on certain sulfates. *An. Stiint. U. Al. I.-Geol.* **55**, 5–23 (2009).

S3. Stammeier, J. A., Purgstaller, B., Hippler, D., Mavromatis, V. Dietzel, M., In-situ Raman spectroscopy of amorphous calcium phosphate to crystalline hydroxyapatite transformation. *MethodsX* **5**, 1241–1250 (2018).

S4. Accorsi, G., Verri, G., Bolognesi, M., Armaroli, N., Clementi, C., Miliani, C. & Romani, A., The exceptional near-infrared luminescence properties of cuprorivaite (Egyptian blue). *Chem. Commun.* **45**, 3392–3394 (2009).

S5. Schmid, T., Kraft, R. & Dariz P., Shedding light onto the spectra of lime – Part 2: Raman spectra of Ca and Mg carbonates and the role of d-block element luminescence. *J. Raman Spectrosc.* **52**, 1462–1472 (2021).

S6. García-Fernández, P., Moreno, M. & Aramburu, J. A., Origin of the anomalous color of Egyptian and Han blue historical pigments: Going beyond the complex approximation in ligand field theory. *J. Chem. Educ.* **93**, 111–117 (2016).

S7. Barrio, R. A., Galeener, F. L., Martínez, E. & Elliott, R. J., Regular ring dynamics in AX_2 tetrahedral glasses. *Phys. Rev. B* **48**, 15672 (1993).

S8. Sriyutha Murthy, P., Venugopalan, V. P., Arunya, D. D., Dhara, S., Pandiyan, R. & Tyagi, A. K., Antibiofilm activity of nano sized CuO. *International Conference on Nanoscience, Engineering and Technology (ICONSET 2011)*, 580–583 (2011).

S9. Rashad, M., Rüsing, M., Berth, G., Lischka, K. & Pawlis, A., CuO and Co_3O_4 Nanoparticles: Synthesis, Characterizations, and Raman Spectroscopy. *J. Nanomater.* **2013**, 714853 (2013).
